# Adaptive self-healing electronic epineurium for chronic bidirectional neural interfaces

**DOI:** 10.1038/s41467-020-18025-3

**Published:** 2020-08-21

**Authors:** Kang-Il Song, Hyunseon Seo, Duhwan Seong, Seunghoe Kim, Ki Jun Yu, Yu-Chan Kim, Jinseok Kim, Seok Joon Kwon, Hyung-Seop Han, Inchan Youn, Hyojin Lee, Donghee Son

**Affiliations:** 1 Biomedical Research Institute, Korea Institute of Science and Technology, Seoul, 02792 Republic of Korea; 2 Medical Device Development Center, Daegu-Gyeongbuk Medical Innovation Foundation, Daegu, 41061 Republic of Korea; 3 School of Medicine, Sungkyunkwan University, Suwon, 16419 Republic of Korea; 4 Department of Electrical and Computer Engineering, Sungkyunkwan University, Suwon, 16419 Republic of Korea; 5 School of Electrical and Electronic Engineering, Yonsei University, Seoul, 03722 Republic of Korea; 6 Nanophotonics Research Center, Korea Institute of Science and Technology, Seoul, 02792 Republic of Korea; 7 KHU-KIST Department of Converging Science and Technology, Seoul, 02447 Republic of Korea; 8 Division of Bio-Medical Science & Technology, KIST School, Korea University of Science and Technology (UST), Seoul, 02792 Republic of Korea

**Keywords:** Biomedical engineering, Electronic devices, Soft materials

## Abstract

Realizing a clinical-grade electronic medicine for peripheral nerve disorders is challenging owing to the lack of rational material design that mimics the dynamic mechanical nature of peripheral nerves. Electronic medicine should be soft and stretchable, to feasibly allow autonomous mechanical nerve adaptation. Herein, we report a new type of neural interface platform, an adaptive self-healing electronic epineurium (A-SEE), which can form compressive stress-free and strain-insensitive electronics-nerve interfaces and enable facile biofluid-resistant self-locking owing to dynamic stress relaxation and water-proof self-bonding properties of intrinsically stretchable and self-healable insulating/conducting materials, respectively. Specifically, the A-SEE does not need to be sutured or glued when implanted, thereby significantly reducing complexity and the operation time of microneurosurgery. In addition, the autonomous mechanical adaptability of the A-SEE to peripheral nerves can significantly reduce the mechanical mismatch at electronics-nerve interfaces, which minimizes nerve compression-induced immune responses and device failure. Though a small amount of Ag leaked from the A-SEE is observed in vivo (17.03 ppm after 32 weeks of implantation), we successfully achieved a bidirectional neural signal recording and stimulation in a rat sciatic nerve model for 14 weeks. In view of our materials strategy and in vivo feasibility, the mechanically adaptive self-healing neural interface would be considered a new implantable platform for a wide range application of electronic medicine for neurological disorders in the human nervous system.

## Abstract (web summary)

Electronic implantable devices should be soft and stretchable, such that nerves can adapt mechanically and autonomously. Here, the authors present an adaptive self-healing electronic epineurium which can form compressive stress-free and strain-insensitive electronics-nerve interfaces.

## Introduction

A peripheral nerve-electronics interface capable of restoring bidirectional communication system in injured nerves can enable reliable control of prosthetic robots and diagnosis and therapy of neurological disorders^1–8^. The safest and most reliable way to chronically interact with peripheral nerves may be accomplished by combining the following: (i) matching the mechanical moduli of the nerve tissue and electronics, (ii) ensuring strain-insensitive ionic/electronic conductivity, (iii) using biocompatible interfacing materials, and (iv) using a low-invasiveness procedure. As a promising candidate for treating injured nervous systems, peripheral nerve cuff electrodes were developed for neuroprosthetic applications in human studies^7–14^. Specifically, spiral cuffs and flat interface nerve electrodes were shown their effectiveness of nerve function restoration achieved by electrical stimulation^8–12^. The recording of sensory compound action potentials (CAPs) was also achieved by the spiral cuffs and utilized to demonstrate foot drop correction^13,14^. However, a continuous compressive force exerted to the peripheral nerves wrapped by the cuffs may lead to the deformation of fascicles and blockage of interfascicular blood vessels, which limits the effective recovery of sensory and motor functions after the nerve repair^15,16^. Although flexible cuff electrodes have shown their stability and effectiveness in both preclinical^17–20^ and clinical^9,11,12^ applications by alleviating nerve compression in the early stages of implantation, shear forces originating from the mechanical modulus mismatch at the electronics-nerve interface can cause severe immune responses that are accompanied by fibrotic tissue formation. Critical issues may then arise such as scar tissue aggravating the peripheral nerve compression caused by the electronic material. This compression-induced scar tissue leads to disconnected ionic pathways between the fasciculus and electronic materials, resulting in measurement malfunctions^18^.

Soft, stretchable electronic materials have attracted much attention as a promising neural platform owing to the following advantages: (i) their mechanical modulus can be manipulated to match that of the target nerve tissues to prevent undesirable compressions, and (ii) their electronic performances can be maintained over a wide range of compressive and tensile strains^21–27^. These advantages have revolutionized intimate neural interface technologies that can be applied to diverse organs in the central and peripheral nervous systems. However, achieving stable interfaces of the bioelectronic devices with the peripheral nerves is still challenging due to mechanically dynamic environment of peripheral nerves induced by repetitive movement of joints and the surrounding muscles^1,16,28^. Recently, Bao et al. reported the fabrication of an intrinsically stretchable hydrogel-based conductor with low interfacial modulus and impedance for reliable neuromodulation, which showed the importance of achieving a mechanical modulus match^29^. This strategy is expected to improve the chronic neural recording capabilities of nerve cuffs. However, it should be accompanied by a complete understanding of how continuous deformation and growth of peripheral nerves over long timescales impact environments ranging from interstitial/extracellular to epineurium–electronics interfaces. Although a recent report showed that strain-induced stress relaxation of the extracellular matrix is highly relevant to biochemical signaling at cellular levels^30^, study for achieving the stress-free peripheral neural interfaces in vivo is still insufficient.

Here, we report a new material strategy for the fabrication of an adaptive, self-healing electronic epineurium (A-SEE) for chronic bidirectional neural interfaces. Our material contains a dynamically crosslinked, tough self-healing polymer (SHP) matrix that efficiently dissipates muscle contraction/relaxation and/or nerve stretching/twisting induced strain energies, thereby alleviating the irreversible damage caused by nerve compression during implantation. Furthermore, water-proof self-bonding properties of the materials enable biofluid-resistant self-locking, which provides conformal nerve-electronics interfaces without using any additional fixation tools^31^ and thereby facilitates efficient and low risk implantation process. We successfully achieve long-term peripheral neural recording and stimulation in awake rats (for 7 weeks). Furthermore, we perform a demonstration of peripheral nerve-to-nerve interfacing for showing high potential of the A-SEE in neuroprosthetic applications. We implant two self-healing neural devices on each of two sciatic nerves in an anesthetized rat, and then transmit feedback neural signals recorded from one side of the sciatic nerve to the other. Our new material and its in vivo feasibility could lead to future clinical electronic medicine for neurological disorders.

## Results

### A-SEE fabrication and its nerve tissue modulus adaptability

**Fig. 1:**
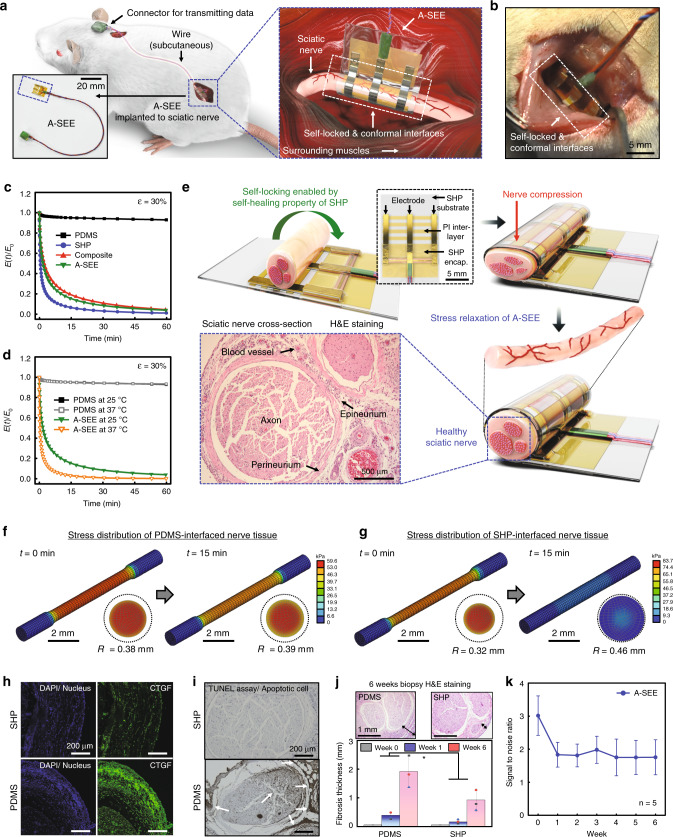
A-SEE and its nerve tissue modulus adaptability. Schematic (**a**) and photograph (**b**) show the A-SEE interfaced with a rat sciatic nerve. **c** Time-dependent normalized tensile stress relaxation of different materials at 30% strain. **d** Temperature dependence of stress relaxation in PDMS and A-SEE. **e** Schematic and H&E stained image of rat sciatic nerve tissue showing the effect of tissue modulus-adaptability of A-SEE on the nerve. Representative image from *n* = 3 biologically independent samples. FEA results showing the time-dependent distributions of compressive stress applied to the nerve interfaced with PDMS (**f**) and SHP (**g**). Confocal fluorescence (**h**) and TUNEL staining (**i**) images of sciatic nerve cross-sections. Representative images from *n* = 3 biologically independent samples. **j**, Thickness of fibrotic tissue formed around the nerve tissues interfaced without (week 0) and with PDMS or SHP for 1 and 6 weeks. Statistical analysis was performed using one-way analysis of variance (ANOVA) with Tukey’s multiple comparison test (*n* = 3 independent samples for each group, *P*(Week 1) = 0.0097, *P*(Week 6) = 0.0104, *P < 0.05). Top inset shows H&E stained images showing the fibrotic tissue formed around the nerve tissue. Representative images from *n* = 3 biologically independent samples. **k** SNR of sensory neural signals recorded by the A-SEE for 6 weeks (*n* = 5 independent animals). In, **j**, **k**, all data are represented as mean ± S.D. Source data are provided as a Source Data file.

A-SEE, composed of tough (SHP, polydimethylsiloxane (PDMS)-4,4’-methylenebis(phenyl urea) (MPU)_0.4_-isophorone bisurea units (IU)_0.6_)^32^ substrate/encapsulation layers and Ag flake-SHP composite (here after referred to as “composite”) electrodes^33^, was realized using self-bonding, a unique property of self-healing materials, which allows individual substrates, electrodes/interconnects, and encapsulation layers to be homogeneously bonded (Supplementary Fig. 1; see Methods for details). As a facile biofluid-resistant self-locking process enabled by the water-proof self-healability of the A-SEE^32^, the self-bonding assembly of the A-SEE does not need additional fixation tools, such as suturing and glue, or closing mechanisms during its implantation (Fig. 1a, b and Supplementary Movie 1). The self-locked A-SEE can form and maintain a conformal interface with the peripheral nerve, which is key for chronic neural interfacing (Fig. 1b and Supplementary Fig. 2).

In addition to its efficient assembly process, A-SEE exhibited compressive stress-free and strain-insensitive performances at the nerve-electronics interfaces such that the strain energy is efficiently dissipated by a dynamically crosslinked SHP matrix including multivalent bonding strengths and dynamic stress relaxation (Fig. 1c, d, and Supplementary Figs. 3, 4)^32,33^. Dynamic mechanical analysis (DMA) results confirm that the tensile stress applied to the SHP, the composite, and the A-SEE can make them extremely relaxed in comparison to PDMS, which was used as a control material as it has a polymeric chain structure and mechanical stiffness similar to those of SHP (Fig. 1c and Supplementary Fig. 5)^29^. This relaxation performance was further enhanced by increasing the temperature (Fig. 1d and Supplementary Fig. 4). Moreover, ex vivo measurement of shear stress at the SHP-nerve tissue interface supported the minimized mechanical mismatches (Supplementary Fig. 6). Specifically, the adaptability of the A-SEE to the nerve tissue modulus based on stress relaxation is capable of significantly reducing the mechanical modulus mismatch at the electronics-nerve interface, which makes A-SEE more suitable for achieving chronic bidirectional peripheral neural interfaces (Fig. 1e). A simplified three-dimensional finite element analysis (FEA) model of PDMS- and SHP-interfaced nerve tissues further confirmed the mechanical adaptability of the A-SEE by demonstrating compressive stress-free SHP-nerve tissue interfaces (Fig. 1f, g, Supplementary Fig. 7, and Table 1).

The sciatic nerve of a rat interfaced with our SHP for 1 week maintained its original structure as well as low immune responses as revealed by low levels of activated macrophages (CD68)^34^, while that interfaced with PDMS showed a compressive-stress induced deformation of the nerve constructs (Fig. 1e and Supplementary Figs. 8-10; see Methods). To further verify the stress-free performance of the A-SEE at the tissue level, we used the connective tissue growth factor (CTGF) as an indicator of the compressive stress applied to the nerve. Generally, CTGF is overexpressed by hypertension and fibrotic disorders that may accelerate extracellular matrix production, which leads to apoptosis^35^. Notably, the pressure regulatory CTGF level of the nerve tissue interfaced with SHP was significantly lower than that interfaced with PDMS (Fig. 1h and Supplementary Figs. 9, 10). The terminal deoxynucleotidyl transferase dUTP nick end labeling (TUNEL) assay results further supported that the compressive stress-free interfaces achieved by SHP resulted in less nerve tissue damage, compared with the PDMS control (Fig. 1i and Supplementary Figs. 9, 10). Importantly, the adaptive mechanical modulus of SHP could significantly alleviate fibrotic tissue formation for 6 weeks (Fig. 1j). This mechanically dynamic property of the A-SEE led to a successful sensory neural signal measurement with maintaining the signal-to-noise ratio (SNR) of about 1.76 even after 6-weeks implantation in sciatic nerves in rats (*n* = 5) (Fig. 1k), which means that the control of interfacial fibrotic tissue growth is key in successful chronic applications.

### Characterization of Au nanomembrane-transferred Ag flake-SHP composite as the A-SEE

**Fig. 2:**
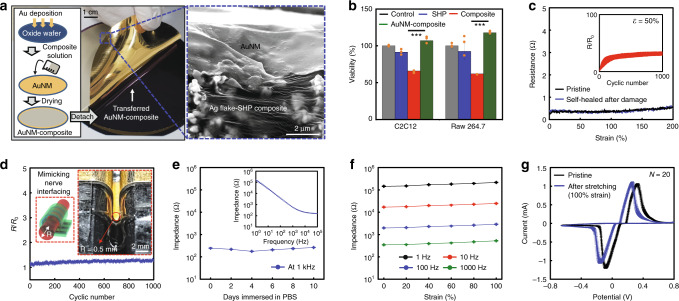
AuNM-Ag flake-SHP composite as a nerve interfacing electrode for the A-SEE. **a** Photograph of AuNM-Ag flake-SHP composite, a schematic showing the transfer-printing of the AuNM onto the Ag flake-SHP composite, and an SEM image showing conformal interfaces between the transferred AuNM and the Ag flake-SHP composite. Representative images from *n* = 5 independent samples. **b** Cell viability test after 7 days culture. Data are represented as mean ± S.D. Statistical analysis was performed using one-way ANOVA with Tukey’s multiple comparison test (*n* = 10^4^ cells/mL examined over five independent experiments, *P*(C2C12) = 0.0001, *P*(Raw 264.7) = 0.0004, ***P < 0.001). **c** Resistance-strain characteristics of the A-SEE in pristine and self-healed states. (Inset) Resistance changes in the A-SEE during a cyclic stretching test at 50% strain for 1000 cycles. **d** Resistance changes in the A-SEE in bending tests that mimic nerve interfacing. (Inset) Photograph showing the A-SEE bent to a bending radius (0.5 mm) of a rat sciatic nerve. **e** Impedance stability of the A-SEE immersed in PBS solution for 10 days. (Inset) Impedance-frequency characteristics of the A-SEE. **f** Strain-dependent impedance of the A-SEE at different frequencies. **g** Cyclic voltammetry curves of the A-SEE at 0 and 100% strain (*n* = 20). Source data are provided as a Source Data file.

The fabrication of the A-SEE consisted of the self-bonding assembly followed by drop-casting an Ag flake-SHP composite in a chloroform solution onto an Au nanomembrane (AuNM) and drying the solvent at room temperature and releasing from the substrate through a weak interaction between Au and silicon dioxide (Fig. 2a and Supplementary Fig. 11). The AuNM-composite structure improves biocompatibility by reducing the cellular toxicity induced by any Ag ions that may have leaked from the composite^36,37^. To verify this, we tested the viability using two different cell lines, immune cells (RAW 264.7) and myoblasts (C2C12), for a week as they interacted with the electrode after implantation in a sciatic nerve (Fig. 2b)^38^. The results show that both cell lines seeded on the AuNM-composite showed excellent viability among the prepared samples, which indicates that AuNM is capable of effectively preventing the Ag-ion leakage. However, the repetitive movement of the rat could induce the formation of micro cracks in AuNM after implantation and the generated cracks may increase the Ag-ion leakage due to the exposed composite surfaces. Therefore, we verified the effect of the micro cracks of AuNM in the strained AuNM-composites (up to 100% stretched) on the cells, and the result showed no cytotoxicity (Supplementary Fig. 12). The amount of Ag-ion leakage in physiological condition was measured directly by inductively coupled plasma-mass spectrometry (ICP-MS), and the results further supported the positive influence of the AuNM on the biocompatibility of the A-SEE (Supplementary Fig. 13). However, the AuNM could not completely prevent the Ag-ion leakage from the composite. To further improve the biocompatibility to overcome the Ag-ion leakage issue, an additional materials strategy of covering individual Ag flakes with the Au shell can be adopted in the future^37^.

Interestingly, the AuNM-composite fabricated using our unique transfer assembly showed better electrical and mechanical characteristics than those of a directly deposited AuNM-composite due to the high interfacial stability of the transferred AuNM and thermal degradation of the deposited AuNM-composite during the deposition process (Supplementary Figs. 14, 15)^33^. Resistance-strain characteristics of the AuNM-composite showed that its electrical performance could be stably maintained during stretching (Fig. 2c). This phenomenon could be explained by the dynamic movement of conducting particles in the strained SHP matrix^33^. Although the AuNM is not self-healing, exceptional stretching performance is exhibited in the self-healed AuNM-composite conductor owing to the dynamic nature of SHP, which is in good agreement with our previous reports (Fig. 2c and Supplementary Fig. 16)^33,39^. The stretching cycle endurance (1000 cycles) under a tensile strain of 50% was also confirmed (Fig. 2c, inset). The mechanical durability of the AuNM-composite contributed to the reliable electrical performance of a folded A-SEE in a nerve-electronics mimicking model (Fig. 2d and Supplementary Fig. 17). The AuNM-composite self-bonded on the SHP substrate showed negligible changes in resistance though it was repetitively bent to a bending radius (0.5 mm) of a rat sciatic nerve.

Furthermore, the electrochemical impedance of the A-SEE at 1 kHz remained constant when immersed in a phosphate buffered saline (PBS) solution for 10 days (Fig. 2e). In addition, the A-SEE could be stretched to 100% strain while maintaining stable electrochemical impedance values (Fig. 2f). Specifically, the A-SEE with low impedance values ranging from 347.6 Ω under 0% strain to 526.5 Ω under 100% strain at 1 kHz may contribute to form stable bidirectional neural interfaces. For confirming its neural stimulation capability, cyclic voltammetry (CV) was performed under 100% strain, which showed the electrochemical stability of the A-SEE (Fig. 2g and Supplementary Fig. 12d). Slight shift of the CV curves may result from the change in surface coverage of the AuNM-composite electrode due to the cracks generated in the AuNM and resultantly exposed Ag flake-SHP composite (see Supplementary Fig. 12d for details).

### Chronic neural recording and stimulation

**Fig. 3:**
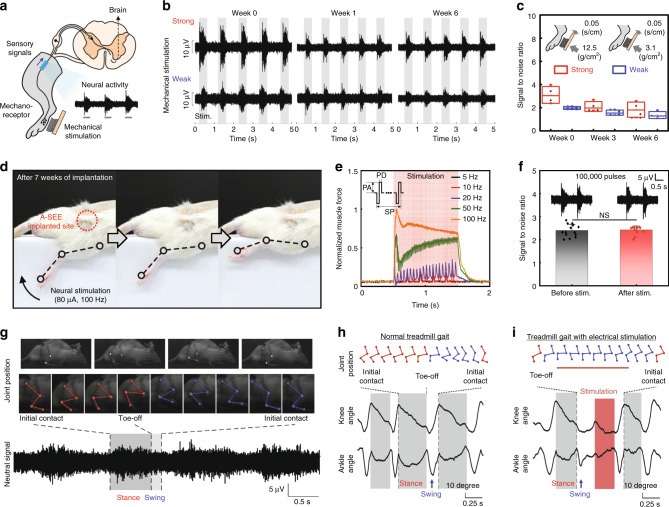
Chronic neural recording and stimulation using the A-SEE. **a** Schematic of sensory neural signal recording during mechanical stimulation. **b** Long-term neural signal recordings with strong and weak mechanical stimuli. **c** SNR of neural signals with strong and weak stimuli at 0, 3, and 6 weeks of implantation (*n* = 5 independent experiments). The centerline represents the median, the bounds of the box represent the upper and lower quartiles, the whiskers represent minimum and maximum values, and the hollow circle shows dot plots. Error bars represent S.D. **d** Sequential images for hind limb movement induced by electrical stimulation (80 μA, 100 Hz) at 7 weeks of implantation. **e** Plot of normalized muscle force as a function of stimulation frequency. The parameters involved in neural stimulation are pulse width (PW, 0.25 ms), biphasic pulse amplitude (PA, 80 μA), stimulation period (0.5 s), repetition stimulation periods (SP, 5, 10, 20, 50, and 100 Hz), and stimulation current (1.14 μC/cm^2^). **f** Changes in SNR performance before and after electrical stimulation (stimulation pulses: 100,000). Data are represented as mean ± S.D. Statistical analysis was performed using one-way ANOVA with Tukey’s multiple comparison test (*n* = 14 independent experiments, *P* = 0.7795, NS (not significant) *P* > 0.05). **g** Synchronous neural signal and joint position recording of treadmill gait after 7 weeks of implantation. Real-time analysis for joint position and knee/ankle angle changes of a live rat without (**h**) and with (**i**) electrical stimulation after 7 weeks of implantation. Source data are provided as a Source Data file.

Realizing a chronic stable recording and stimulation in peripheral or central nervous systems is crucial to treating neurological disorders (Fig. 3a). To understand the signaling complexity of such nervous systems, we implanted the A-SEEs in sciatic nerves in rats (*n* = 5) to measure the CAPs generated by strong (0.12 N/cm^2^) and weak (0.03 N/cm^2^) mechanical stimuli (Fig. 3b and Supplementary Fig. 18; see “Methods”). To mimic neurotransmission, we directly recorded the neural signals induced by mechanoreceptors rather than the measurements of specific waveforms evoked by electrical stimulation using additional stimulation electrodes^19,20^. Although the CAP amplitudes decreased in the early stage of implantation (7 days), we could verify the strong or weak stimulation for 6 weeks owing to the stable mechanical and electrical functionalities of the A-SEE (Figs. 1i, 3c, and Supplementary Figs. 19, 20). The decrease in SNR after the first week (Figs. 1i and 3c) is mainly because the immune responses occur undesirably during the invasive implantation surgery^18^. However, such recorded sensory neural signals can be further clarified by the denoising process (Supplementary Fig. 21 and Supplementary Table 2; see “Methods”). As shown in Supplementary Fig. 21, it is evident that the present A-SEE exhibits stable and long-term (i.e., 6 weeks) neural signal detection performance. The results suggest that our A-SEE was able to minimize immune responses by adapting its modulus to the nerve tissue and was thus suitable as a stable chronic neural interfacing system in vivo (Supplementary Fig. 22).

Furthermore, the levels of CD68 and CTGF in the nerves implanted with the A-SEE for 6 weeks were almost similar to the protein level for 1 week, which supports such chronic reliable performances (Supplementary Figs. 22, 23)^40^. Although the protruding structures of the electrodes in the A-SEE resulted in a small pointed fibrosis due to the physical height gap between parts of the A-SEE and the nerve, we confirmed that the A-SEE with the modified design having in-plane electrodes could also achieve chronic stable neural signal recordings without showing any fibrotic tissue formation (Supplementary Fig. 24). Neural stimulation was also performed (Fig. 3d and Supplementary Movie 2) by monitoring the quantitative force values induced by applying various electrical stimulations to the rat sciatic nerve for 7 weeks (Fig. 3e). The result indicated that a large muscle force was elicited by electrical stimulation at a high repeated frequency in contrast with a low repeated frequency, and the tetanic and twitch muscle forces were observed at high stimulation frequencies (higher than 20 Hz) and low stimulation frequencies (below 20 Hz,) respectively. These results almost matched with those in the previous report^41^. Moreover, even after repetitive electrical stimulations (over 100,000 pulses) were applied to the A-SEE, the neural recording performance was stably maintained due to robust adhesion between the AuNM and the composite (Fig. 3f). ICP-MS analysis of biopsy samples obtained from rats after the 6-week A-SEE implantation supported these results (Supplementary Table 3).

Figure 3g depicts an example of neural signaling and joint kinematics from a live rat during treadmill walking (Supplementary Fig. 25 and Supplementary Movie 3). The gait cycles were divided into two phases: stance (red) and swing (blue). The stance phase was defined as the part of the gait cycle from the initial contact to the toe-off; the swing phase was defined as the part of the gait cycle from toe-off to the next initial contact. The neural signal could be classified synchronously with each phase from joint kinematics. Interestingly, real-time analyses of joint positions and knee/ankle angle changes showed that the gait could be successfully modulated and controlled by applying a specific stimulation (Fig. 3h, i). Furthermore, even after the abovementioned repetitive electrical stimulation for neuromodulation and joint kinematic analysis, the sensory neural signals could be clearly recorded at 14 weeks of implantation (Supplementary Fig. 26 and Supplementary Movie 4). Notably, we also confirmed reliable performance of neural stimulations using the A-SEE after 32 weeks of implantation (Supplementary Fig. 27 and Supplementary Table 3). These results indicate that A-SEE can stably interface with the nerve tissue without any device failure and also maintain the health and functions of the sciatic nerve for extended periods.

### Peripheral nerve-to-nerve interfacing demonstration

**Fig. 4:**
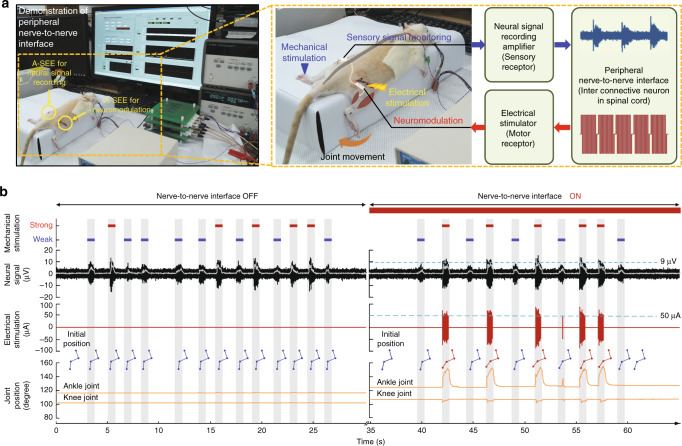
Nerve-to-nerve interface. **a** Experimental set-up and flow diagram of nerve-to-nerve interfacing demonstration. **b** Transmission of neural signals recorded from one side of sciatic nerve to the other through nerve-to-nerve interface.

Such bidirectional neural recording and stimulation performance enabled an interesting demonstration of peripheral nerve-to-nerve interfacing for showing the potential of the A-SEE as a neuroprosthetic device platform. (Fig. 4a and Supplementary Movie 5; see “Methods”). We implanted an A-SEE in each sciatic nerve of the anesthetized rat, and each A-SEE was linked with a home-made controller system, which could transmit feedback neural signals recorded from one side of the sciatic nerve to the other (Fig. 4b; see “Methods”). Specifically, the evoked CAPs in the ipsilateral nerve originate from the mechanical stimulation of the limb using the brush. After analyzing the recorded signals from the system, we can induce movement of the contralateral limb through the electrical stimulation.

Using the nerve-to-nerve interface system based on A-SEE, we demonstrated a behavior similar to the crossed extensor reflex. Specifically, in the designed system, the functions of neural signal amplifier, electrical stimulator, and nerve-to-nerve controller correspond to sensory receptor, motor receptor, and inter connective neuron in spinal cord, respectively. When the amplitude of tactile sensory neural signals recorded from the ipsilateral nerve is above threshold that we programmed, electrical stimulation of the contralateral nerve is triggered and thereby inducing the movement of the contralateral limb (right, Fig. 4b). Although this application did not perfectly follow the biotic crossed extensor reflex pathway, it performed a similar function, generated muscle contractions from sensory stimuli, and linked sensory functions from the ipsilateral site with motor functions in the contralateral site. The interactive peripheral nerve-to-nerve interfacing technology would be helpful to improve future clinical electronic medicine or neuroprosthetics^42,43^.

## Discussion

Peripheral nerve cuff electrodes have proven their clinical efficacy as a desirable implantable platform for neuroprosthetic applications. However, nerve compression unavoidably caused by mechanical mismatch between nerve tissues and the implanted electronics still remains as a critical issue that hampers further progress in the cuff-type neural interfacing technology. To address the issue, we present a new materials and device strategy for realizing soft, conformal, and compressive stress-free peripheral nerve-electronics interfaces by utilizing SHP-based materials design. Along with the softness and high stretchability, excellent self-locking capability of the SHP allows to easily form soft, conformal interface without requiring any additional fixation process and with avoiding irreversible damages on nerve. More importantly, efficient stress relaxation of SHP confers unique mechanical adaptive property to the electronics that enables significant mitigation of compressive stress applied to the nerve. Owing to such materials properties, we successfully designed A-SEE that achieves stable bidirectional neural recording and stimulation in a rat sciatic nerve for 7 weeks. The demonstration shows a promising potential to improve the clinical utility of peripheral neural interfaces. We envision that the A-SEE would be pursued in clinic for chronical treatment of neurological disorders.

## Methods

### Materials for adaptive self-healing electronic epineurium (A-SEE)

Synthesis of SHP and Ag flake-SHP composite followed our previous reports^32,33^. The Ag flake-SHP composite with a weight ratio of 4:1 (Ag flake:SHP) was used in this study. Two different methods were tried to introduce AuNM to the Ag flake-SHP composite: (1) transfer and (2) direct deposition. AuNM of 60 nm thickness was first deposited on a SiO_2_ wafer using an e-beam evaporator (ULVAC Co.). Then, a Ag flake-SHP mixed chloroform solution with an appropriate viscosity^33^ was poured onto the AuNM-deposited wafer and left to dry at room temperature for 12 h. When the sample was dry, the AuNM-composite was easily detached from the wafer. This process resulted in the conformal transfer of the AuNM from the SiO_2_ wafer due to the relatively strong Au–Ag and Au–polymer interactions compared with the Au–SiO_2_ interaction. In the second method, the AuNM was directly deposited to the top surface of the composite supported on an octadecyltrimethoxysilane-treated silicon wafer. After comparing the mechanical and electrical performances of the transferred AuNM-composite and those of the directly deposited AuNM-composite, we used the transferred AuNM-composite for further experimentation. The fabrication process of the A-SEE based on self-bonding assembly using SHP and the AuNM-composite is explained in Supplementary Fig. 1.

For the interconnection between A-SEE and neural signal recording amplifier, a Au pad with a PI substrate was used. Each pad had a 1 mm width and a 4 mm length with a hole for self-bonding between the AuNM-composite and the SHP substrate (hole dimensions (w × l): 0.35 mm × 2 mm), and was composed of a PI, Ti, Cu, and Au layer of 80, 5, 30, and 10 nm thickness, respectively. After the self-bonding of the Au pad with the AuNM-composite layer and SHP substrate layer, a Teflon-coated wire was soldered onto the other end of the Au for connecting with the neural signal recording amplifier. Silicone casting compound (Kwik-cast, World precision instruments) was used for encapsulating the soldered wire.

### Characterizations of A-SHE

#### SEM analysis

Field emission scanning electron microscopy (Inspect F50, FEI Co.) was used to observe the interfaces between the composite and the transferred or directly deposited AuNM. Each AuNM- composite film was cross-sectioned by using a razor blade and coated by platinum before imaging.

#### Stress relaxation characterization

For DMA analysis, the 0.3 mm thick SHP film, the 0.3 mm thick AuNM-composite film, the 0.9 mm thick encapsulated AuNM-composite (A-SEE) film, and the 0.3 mm thick PDMS (Sylgard 184, 20:1 PDMS:crosslinking agent weight ratio) film were each cut to have a length of 10 mm and a width of 5 mm. We chose PDMS with a 20:1 ratio as the control material in all experiments due to its value of Young’s modulus (131.8 ± 13.8 kPa), which is comparable with that of SHP (163.3 ± 42.1 kPa) (Supplementary Fig. 5). Then, each sample was stretched to a tensile strain of 30% with a stretching speed of 50% strain per minute and the tensile stress at the strain was measured for 60 min by DMA Q800 (TA Instruments). The stress relaxation properties of SHP, PDMS, composite, and the A-SEE were measured at two different temperatures (25 °C and 37 °C) to determine the temperature dependence.

#### Electrical characterization

For resistance-strain characterization, each AuNM-composite (3 mm length, 5 mm width, 0.3 mm thickness) self-bonded to the SHP substrate (3 mm length, 7 mm width, 0.2 mm thickness) (A-SEE) was stretched by using an automatic stretching machine with a stretching speed of 100% strain per minute and the resistance was measured using a four-point probe method (Keithley 2400, Tektronix). For the cyclic stretching test, a strain of 50% was repeatedly applied for 1000 cycles. For the nerve interfacing mimicking test, the AuNM-composite with a length of 10 mm, a width of 5 mm, and a thickness of 0.3 mm was repeatedly bent to reach a bending radius of 0.5 mm for 1000 cycles. For the self-healing test, the sample was completely cut using a razor blade and then self-healed by being heated at 60 °C for 1.5 h.

#### Electrochemical characterization

Electrochemical impedance and CV were measured using a potentiostat (CHI 760 C, CH Instruments) in the PBS solution. The exposed area of the samples was 7.5 mm2 and a platinum wire was used as the counter electrode in both measurements. Potentiostatic electrochemical impedance spectroscopy was conducted with a frequency ranging from 1 Hz to 1 MHz and an amplitude of 10 mV. For the CV measurement, an Ag/AgCl electrode (BASiAg/AgCl/3 M NaCl) was used as the reference electrode, and the curves were obtained from −0.65 V to 0.8 V at a scan rate of 100 mV/s. The charge delivery capacity was calculated using the following Eq. (1).

1 $${\mathrm{CDC}} = \frac{1}{{vA}}\int_{Ec}^{Ea} \left| i \right|dE\left( {C/cm^2} \right).$$

Here, *v, A, E*_*a*_*, E*_*c*_*, i*, and *E* are scan rate (V/s), geometrical surface area of the electrode (cm^2^), anodic/cathodic potential limit (V), measured current, and electrode potential (V vs Ag/AgCl), respectively.

### Finite element analysis

A simplified three-dimensional model of PDMS- and SHP-interfaced nerve tissues was created for FEA using ANSYS Workbench (Release 16.1; ANSYS Inc. Canonsburg, PA). The simplified three-dimensional model contained 23,216 nodes and 4530 elements and was assumed to be bonded at contacting surfaces, homogeneous, isotropic, and linearly elastic. The mechanical properties of the nerve tissue and the materials were determined and are listed in Supplementary Table 1. The nerve tissue was assumed to be cylindrical with a 1 mm diameter and 10 mm height. The tested materials were also assumed to be hollow cylinders with 1 mm inner diameter, 1.6 mm outer diameter, and 5 mm height that were wrapped around the nerve tissue. The stress measured from the DMA results at a specified time point, shown in Supplementary Fig. 3 and summarized in Supplementary Table 1, was applied as pressure on the outer layer of the tested material to determine the resulting stress distribution on the nerve tissue. The two-dimensional cross-sectional distributions were obtained as a cross-section of the center of the nerve tissue. The dotted circles indicate the original diameter of the nerve tissue before it was interfaced with the materials.

### In vitro biocompatibility test

RAW 264.7 and C2C12 cell lines were purchased from American Type Culture Collection and were cultured in Dulbecco’s Modified Eagle’s Medium (DMEM) with 10% fetal bovine serum at 37 °C. For testing the biocompatibility of the materials (SHP, composite, and AuNM-composite), the cells were seeded in 96-well at a concentration of 1 × 10^4^ cells/mL. After 7 days of incubation of cells with the same size of each material, the cell viability was measured using an MTS assay solution (G3585, Promega, Wisconsin, USA). The absorbance of the reacted solution at a wavelength of 490 nm was recorded using a 96-well plate reader (Infinite 200 Pro, Tecan, Männedorf, Switzerland).

### Sterilization and preparation for the animal study

The SHP, PDMS, and A-SEE samples were immersed in 70% ethanol solution and washed twice with PBS solution before implantation to prevent contamination and remove the PDMS precursor (see Supplementary Fig. 8)^44,45^.

### Animal preparation and implantation of A-SEE

All animal experiments were performed and handled per the regulation of the Institutional Animal Care and Use Committee of the Korea Institute of Science and Technology (Approval No. 2018-067). The experimental procedure was performed according to the Guide for the Care and Use of Laboratory Animals^46^. For the A-SEE implantation, a Sprague-Dawley rat (male, 300 g) was anesthetized using an intraperitoneal injection of 50 mg/kg Zoletil and 10 mg/kg Xylazine mixture. After a deep level of anesthesia was achieved, the skin incision was extended to the dorsal aspect of the paw to expose the hind limb musculature. The lateralis and biceps femoris muscles were then identified and retracted to expose the sciatic nerve. Then the A-SEE was wrapped around the sciatic nerve after the surrounding tissue was removed. Self-locking process for wrapping the nerve takes within a minute in general. During the process, we just need to maintain contact between the folded SHP films (substrate of A-SEE) by using surgical tweezers for a while. After completing the self-locking, the self-bonded A-SEE is difficult to separate again unless it is amputated using scalpel. However, since the contacted SHP films can be detached within few seconds, careful adjustment of position or posture of the A-SEE is possible during the implantation.

### In vivo nerve compression comparison of SHP with PDMS

We prepared PDMS and SHP cuff structures through a molding process. Both PDMS and SHP cuff structures have an inner diameter of 1 mm (similar to the diameter of the sciatic nerve) and a slit that allows them to be opened and applied to sciatic nerves and then closed autonomously. These were implanted onto the two sciatic nerves to confirm how their mechanical properties affect the nerves for a week. After implanting the PDMS cuff structure to the nerve, it was locked using silicone casting compound (Kwik-cast, World precision instruments). The SHP cuff structure was locked by the self-bonding assembly. After 1 week of implantation, each of the sciatic nerves were investigated using histological and immunofluorescence analyses (see Supplementary Fig. 9).

### Neural signal data acquisition

For neural signal recording, a nerve cuff electrode system that was developed in our previous report^47^ was used (see Supplementary Fig. 18). This electrode system was composed of a preamplifier and an external module with a 39,601 gain and a −3 dB bandwidth from 425 to 5500 Hz. An A-SEE interfaced with a rat sciatic nerve was connected to a preamplifier and the final output of the external amplifier was digitized using an analog-to-digital converter board (PXI-6733, National Instrument, USA) with a sampling frequency of 25 kHz. Then, the acquired signals were processed using the LabVIEW software (National Instruments, USA).

For neural signal recording in a moving animal, the preamplifier was implanted in the subcutaneous layer of a rat’s back with a transcutaneous head connector. The neural signal and the two-dimensional motion data were recorded synchronously. The motion system was composed of a high speed digital camera (Marlin F033B, AVT, Germany) and grabber board with a 640 × 480 pixel resolution. The digital camera was positioned perpendicular to the walking trace and the images were recorded at 60 frames/s. The anatomical landmarks were defined to the lateral side of the hind limb at the knee, ankle, and fifth metatarsal joint^41^.

### In vivo neural signal recording

The in vivo neural signal recording was performed for 7 weeks with the right leg of five animals. The animals were anesthetized with 50 mg/kg Zoletil and 10 mg/kg Xylazine mixture by the intraperitoneal injection. The glabrous skin of the right hind paw was mechanically stimulated by using a pig hair brush, and the stimuli were induced with two different pressure intensities. The stimuli were classified as strong and weak, which correspond to 12.5 g/cm^2^ (220 g–16 cm^2^) at 0.05 s/cm and 3.1 g/cm^2^ (50 g–16 cm^2^) at 0.05 s/cm, respectively. Each stimulus was measured by a force gauge (Mark-10, USA). Each mechanical stimulus lasted ~1 s and was repeated approximately every 5 s. For each trial, the neural signal was recorded for 60 s, and each trial included 10 stimuli. For quantification of the neural signals, the SNR was defined as the ratio of the root mean voltage of the neural signals during the stimulus-present period to that during the stimulus-absent period. For each amplifier, the average SNR was calculated from five trials and each amplifier was used in five rats.

### Denoising process of the recorded neural signals

The recorded neural signals were denoised using a 1D wavelet denoising algorithm^48^. To secure robustness in wavelet transform, we applied a maximal overlap discrete wavelet transform method. In Supplementary Fig. 21, raw, and denoised data were compared with variance change behaviors of the signals as a function of time-window used in the wavelet transform.

### Calculation of SNR ratio between the recorded neural signals under stimulation and at rest state

For time-dependent signal data$S\left( t \right)$, which is a set of $N$ discrete signal points ($S\left( t \right) = \left\{ {s\left( {t_i} \right)} \right\},{\mathrm{ where }}i = 1,2, \cdots ,N$), the residual noise, $N\left( t \right)$, can be expressed as

$$N(t) = S(t) - S_d(t),$$

where $S_d(t)$ denotes the denoised time-dependent signal. Using $N\left( t \right)$, the SNR can be expressed as

$$SNR = 10\log _{10}\left( {\frac{{\left\langle {S\left( t \right)^2} \right\rangle }}{{\left\langle {N\left( t \right)^2} \right\rangle }}} \right),\,\left\langle {f\left( t \right)^2} \right\rangle \equiv \frac{1}{N}\mathop {\sum}\limits_{i = 1}^N {s\left( {t_i} \right)} ^2,$$

To compare SNR difference between two time-dependent signal data, $S_{stim}\left( t \right)$ and $S_{rest}\left( t \right)$, where the subscripts $stim$ and $rest$ denote periods under stimulation and of rest, respectively, we can calculate the relative SNR which can be expressed as

$$SNR_{rel} = SNR_{stim} - SNR_{rest}.$$

Using $SNR_{rel}$, we can quantitatively compare the deterioration dynamics of the neural signal transduction affected by degradation of the flexible electrode. A detailed procedure is provided in Supplementary Table 2.

### In vivo electrical stimulation

An electrical stimulator (AM 2200, AM-system) with a programmed pulse generator, which was developed in our previous report^49^, was used to induce the biphasic current pulse on the sciatic nerve. The biphasic pulse was set to a 0.25 ms pulse width and different repetition stimulation pulse periods (5, 10, 20, 50, and 100 Hz). The stimulation period and current were set to 0.5 s and 1.14 μC/cm^2^, respectively. The amplitude of the stimulation pulse of 80 μA was chosen based on the minimum thresholds for joint movements. The muscle force response from electrical stimulation was measured using a torque sensor (QWFK-8M, Honeywell) with a shoe shaft. The torque data were digitalized using an analog-to-digital converter board (PXI-6143, National instruments). The muscle forces were normalized to the values at frequency 100 Hz.

### Demonstration of nerve-to-nerve interfacing

The nerve-to-nerve interface system was composed of neural signal amplifier, electrical stimulator and nerve-to-nerve controller, which were modified from our previous work^49^. For nerve-to-nerve interfacing demonstration, two A-SEEs were implanted to each ipsilateral and contralateral sciatic nerve, respectively, and each A-SEE interfaced with the ipsilateral and contralateral sciatic nerve performed the neural signal recording and stimulation, respectively. The neural signal recording and electrical stimulation were performed using the same experimental setup as in the in vivo neural signal recording and in vivo electrical stimulation. The recorded neural signals from the ipsilateral sciatic nerve were converted to the stimulation parameter via a nerve-to-nerve controller and the electrical stimulation pulses were applied to the contralateral sciatic nerve. To verify the demonstration of mimicking crossed extensor reflex, the ankle and knee joint position of the contralateral limb were measured during strong and weak mechanical stimulation on the ipsilateral site paw using the brush. The joint position measurement was performed using the same experimental setup as in the in vivo neural signal recording in a moving animal. The joint movements of the contralateral limb were triggered only when the amplitude of sensory neural signals recorded from the ipsilateral nerve was above the threshold that we programmed using a rectified bin integration method^49^. The threshold, the neural signal amplitude of 9 μV, was converted to the electrical stimulation amplitude of 50 μA. The overall system was processed by using LabVIEW software (National Instruments, USA).

### Western blot

After scarification, nerve tissue samples were immersed in tissue protein extraction reagent (T-PERTM, #78510, Thermo Fisher Scientific) at a ratio of 1:20 (w/v) for blotting. The samples were sonicated for 5 min in ice and centrifuged at 13572 g and 4 °C for 15 min. We collected the supernatant and then determined total protein concentration using the PierceTM BCA Protein assay kit (#23225, Thermo Fisher Scientific, #23225) according to the manufacturer’s instructions. Next, 15 µg protein from each sample was loaded on an SDS-PAGE gel (Biorad) and subjected to electrophoresis in a 1X Tris-Glycine SDS buffer (T8053-101, GenDepot) at 130 V for 60 min. The separated proteins were transferred to a nitrocellulose membrane, 0.45 μm (#1620145, Biorad) in 1X Tris-Glycine Native buffer (HT2028, Biosesang) with 10% methanol at 4 °C. To detect the target protein, the membrane was incubated with specific primary antibodies; GAPDH (ab9485, Abcam, 1:2500), CD68 (ab53444, Abcam, 1:1000) and CTGF (ab6992, Abcam, 1:1000). Two types of horse-radish-peroxidase (HRP)-conjugated antibodies, anti-rabbit (ab6721, Abcam, 1:5000) and anti-rat (SC-2006, SantaCruz, 1:5000), were used as a secondary antibody. After a 1-h incubation with 2nd antibodies, the membrane was washed with PBS solution and then was treated with a detection agent (WSE-7120L, ATTO corporation). Finally, the desired bands were imaged by an ImageQuant LAS 4000 mini (GE Healthcare).

### Histology and immunofluorescence analysis

The nerve tissues were fixed in a 4% paraformaldehyde solution (P2031, Biosesang) and embedded in paraffin blocks. The blocks were sliced into 5 μm thick sections with Microtome (RM2255, Leica Biosystem). The paraffin sections were deparaffinized with Xylene (#8587-4400, Daejung) and hydrated with decreasing concentration of alcohol. For tissue histology imaging, the sections were stained with hematoxylin and eosin and terminal deoxynucleotidyl TUNEL assay using standard protocols. The images were monitored using an optical microscope (LAS X Widefield, Leica). For immunostaining, hydrated paraffin sections were blocked with 0.3% H_2_O_2_ for 30 min and soaked in 0.01 M citrate (C0759, Sigma-Aldrich) buffer pH 6.0 at 95 °C for 20 min. The sections were blocked with 3% BSA in PBS solution (#10010023, Gibco) for 1 h and were incubated overnight at 4 °C with the primary antibodies, CD68 (ab53444, Abcam, 1:500) and CTGF (ab6992, Abcam, 1:500). The samples were incubated with the secondary antibodies, a Donkey Anti-Rat IgG Alexa Fluor® 647 (ab150155, Abcam, 1:5000) and Alexa FluorTM 488 goat anti-rabbit IgG (A-11008, Invitrogen, 1:5000), for 1 h at room temperature. The slides were mounted with Vectashield mounting medium with DAPI (H-1200, Vector Laboratories) and were observed using a confocal microscope (LSM 700, Carl Zeiss). For quantification of the obtained immunostaining and TUNEL staining images, the cross-section nerve tissue images were obtained with the same size (1069 × 1069 pixels for immunostaining images and 5184 × 3456 pixels for TUNEL stained images) at the same exposure time and were selected randomly (*n* = 3). Then, the fluorescence intensity of the images was measured using the ImageJ program, and the brightness intensities of the black and white threshold color-inverted TUNEL stained images were quantified. For the relative fluorescence intensity, a mean value of CD68 and CTGF divided by the value of DAPI was used.

### Quantification of Au and Ag-ion leakage

For the in vitro Ag-ion leakage measurement, the composite and the AuNM-composite were incubated in cell culture medium (DMEM, Dulbecco’s Modified Eagle’s Medium) for 7 days, respectively. Then, we collected the culture media to measure the amount of Ag ions and determine the potential Ag-induced toxicity of the composites in physiological conditions. For in vivo quantification, the nerve tissues interfaced with an A-SEE for 6 weeks were digested in an acid mixture containing 30% HCl (Sigma-Aldrich) and 70% HNO_3_ (Sigma-Aldrich) at 75 °C. After the complete digestion of the material, the solution was diluted with Millipore water. The Au and Ag contents were measured using ICP-MS.

### Statistical analysis

The statistical analysis was performed using Origin 2020 software.

### Reporting summary

Further information on experimental design is available in the Nature Research Reporting Summary linked to this paper.

## Supplementary information

Supplementary Information

Description of Additional Supplementary Files

Supplementary Movie 1

Supplementary Movie 2

Supplementary Movie 3

Supplementary Movie 4

Supplementary Movie 5

Reporting Summary

## Source data

Source Data

## Data Availability

The data that support the findings of this study are available from the corresponding author upon reasonable request. Source data are provided as a Source Data file. Source data are provided with this paper.

## References

[1] Lacour, S. P., Courtine, G. & Guck, J. Materials and technologies for soft implantable neuroprostheses. *Nat. Rev. Mater.* **1**, 16063 (2016).

[2] Patil, A. C. & Thakor, N. V. Implantable neurotechnologies: a review of micro- and nanoelectrodes for neural recording. *Med. Biol. Eng. Comput.* **54**, 23–44 (2016). 26753777 10.1007/s11517-015-1430-4

[3] Chen, R., Canales, A. & Anikeeva, P. Neural recording and modulation technologies. *Nat. Rev. Mater.* **2**, 16093 (2017). 31448131 10.1038/natrevmats.2016.93 PMC6707077

[4] Kang, S.-K. et al. Bioresorbable silicon electronic sensors for the brain. *Nature* **530**, 71–76 (2016). 26779949 10.1038/nature16492

[5] Yu, K. J. et al. Bioresorbable silicon electronics for transient spatiotemporal mapping of electrical activity from the cerebral cortex. *Nat. Mater.* **15**, 782–791 (2016). 27088236 10.1038/nmat4624 PMC4919903

[6] Hong, G. et al. A method for single-neuron chronic recording from the retina in awake mice. *Science* **360**, 1447–1451 (2018). 29954976 10.1126/science.aas9160 PMC6047945

[7] Charkhkar, H., Christie, B. P., Pinault, G. J., Tyler, D. J. & Triolo, R. J. A translational framework for peripheral nerve stimulating electrodes: reviewing the journey from concept to clinic. *J. Neurosci. Methods* **328**, 108414 (2019). 31472187 10.1016/j.jneumeth.2019.108414

[8] Tan, D. W. et al. A neural interface provides long-term stable natural touch perception. *Sci. Transl. Med.* **6**, 257ra138 (2014). 25298320 10.1126/scitranslmed.3008669 PMC5517305

[9] Tan, D. W., Schiefer, M. A., Keith, M. W., Anderson, J. R. & Tyler, D. J. Stability and selectivity of a chronic, multi-contact cuff electrode for sensory stimulation in human amputees. *J. Neural Eng.* **12**, 026002 (2015). 25627310 10.1088/1741-2560/12/2/026002 PMC5517311

[10] Delianides, C., Tyler, D. W., Pinault, G., Ansari, R. & Triolo, R. Implanted high density cuff electrodes functionally activate human tibial and peroneal motor units without chronic detriment to peripheral nerve health. Neuromodulation 10.1111/ner.13110. (2020). 10.1111/ner.13110 32189421

[11] Fisher, L. E., Tyler, D. W., Anderson, J. S. & Triolo, R. J. Chronic stability and selectivity of four-contact spiral nerve-cuff electrodes in stimulating the human femoral nerve. *J. Neural Eng.* **6**, 046010 (2009). 19602729 10.1088/1741-2560/6/4/046010 PMC2928075

[12] Christie, B. P. et al. Long-term stability of stimulating spiral nerve cuff electrodes on human peripheral nerves. *J. Neuroeng. Rehabil.* **14**, 70 (2017). 28693584 10.1186/s12984-017-0285-3 PMC5504677

[13] Davis, L. A., Gordon, A., Hoffer, J. A., Jhamandas, J. & Stein, R. B. Compound action potentials recorded from mammalian peripheral nerves following ligation or restoring. *J. Physiol. Paris* **285**, 543–559 (1978). 10.1113/jphysiol.1978.sp012588 PMC1281773 370366

[14] Hoffer, J. A. et al. Neural signals for command control and feedback in functional neuromuscular stimulation: a review. *J. Rehabil. Res. Dev.* **33**, 145–157 (1996). 8724170

[15] Grill, W. M. & Mortimer, J. T. Neural and connective tissue response to long-term implantation of multiple contact nerve cuff electrodes. *J. Biomed. Mater. Res.* **50**, 215–226 (2000). 10679687 10.1002/(sici)1097-4636(200005)50:2<215::aid-jbm17>3.0.co;2-a

[16] Navarro, X. et al. A critical review of interfaces with the peripheral nervous system for the control of neuroprostheses and hybrid bionic systems. *J. Peripher. Nerv. Syst.* **10**, 229–258 (2005). 16221284 10.1111/j.1085-9489.2005.10303.x

[17] Naples, R. G., Mortimer, J. T., Scheiner, A. & Sweeney, J. D. A spiral nerve cuff electrode for peripheral nerve stimulation. *IEEE Trans. Biomed. Eng.* **35**, 905–916 (1988). 3198136 10.1109/10.8670

[18] Thil, M. A., Duy, D. T., Colin, I. M. & Delbeke, J. Time course of tissue remodeling and electrophysiology in the rat sciatic nerve after spiral cuff electrode implantation. *J. Neuroimmunol.* **185**, 103–114 (2007). 17343923 10.1016/j.jneuroim.2007.01.021

[19] Xiang, Z. et al. Progress of flexible electronics in neural interfacing - a self-adaptive non-invasive neural ribbon electrode for small nerves recording. *Adv. Mater.* **28**, 4472–4479 (2016). 26568483 10.1002/adma.201503423

[20] Zhang, Y. et al. Climbing-inspired twining electrodes using shape memory for peripheral nerve stimulation and recording. *Sci. Adv.* **5**, eaaw1066 (2019). 31086809 10.1126/sciadv.aaw1066 PMC6505533

[21] Feiner, R. & Dvir, T. Tissue–electronics interfaces: from implantable devices to engineered tissues. *Nat. Rev. Mater.* **3**, 17076 (2017).

[22] Won, S. M. et al. Recent advances in materials, devices, and systems for neural interfaces. *Adv. Mater.* **30**, e1800534 (2018). 29855089 10.1002/adma.201800534

[23] Minev, I. R. et al. Electronic dura mater for long-term multimodal neural interfaces. *Science* **347**, 159–163 (2015). 25574019 10.1126/science.1260318

[24] Rivnay, J., Wang, H., Fenno, L., Deisseroth, K. & Malliaras, G. G. Next-generation probes, particles, and proteins for neural interfacing. *Sci. Adv.* **3**, e1601649 (2017). 28630894 10.1126/sciadv.1601649 PMC5466371

[25] Lee, W. et al. Nonthrombogenic, stretchable, active multielectrode array for electroanatomical mapping. *Sci. Adv.* **4**, eaau2426 (2018). 30345362 10.1126/sciadv.aau2426 PMC6195340

[26] Koo, J. et al. Wireless bioresorbable electronic system enables sustained nonpharmacological neuroregenerative therapy. *Nat. Med.* **24**, 1830–1836 (2018). 30297910 10.1038/s41591-018-0196-2

[27] Hong, G. & Lieber, C. M. Novel electrode technologies for neural recordings. *Nat. Rev. Neurosci.* **20**, 330–345 (2019). 30833706 10.1038/s41583-019-0140-6 PMC6531316

[28] Topp, K. S. & Boyd, B. S. Structure and biomechanics of peripheral nerves: nerve responses to physical stresses and implications for physical therapist practice. *Phys. Ther.* **86**, 92–109 (2006). 16386065 10.1093/ptj/86.1.92

[29] Liu, Y. et al. Soft and elastic hydrogel-based microelectronics for localized low-voltage neuromodulation. *Nat. Biomed. Eng.* **3**, 58–68 (2019). 30932073 10.1038/s41551-018-0335-6

[30] Nam, S., Hu, K. H., Butte, M. J. & Chaudhuri, O. Strain-enhanced stress relaxation impacts nonlinear elasticity in collagen gels. *Proc. Natl Acad. Sci. USA.* **113**, 5492–5497 (2016). 27140623 10.1073/pnas.1523906113 PMC4878492

[31] Rijnbeek, E. H., Eleveld, N. & Olthuis, W. Update on peripheral nerve electrodes for closed-loop neuroprosthetics. *Front. Neurosci.* **12**, 350 (2018). 29910705 10.3389/fnins.2018.00350 PMC5992394

[32] Kang, J. et al. Tough and water-insensitive self-healing elastomer for robust electronic skin. *Adv. Mater.* **30**, e1706846 (2018). 29424026 10.1002/adma.201706846

[33] Kim, S. H. et al. An ultrastretchable and self-healable nanocomposite conductor enabled by autonomously percolative electrical pathways. *ACS Nano* **13**, 6531–6539 (2019). 31072094 10.1021/acsnano.9b00160

[34] van Putten, S. M., Ploeger, D. T., Popa, E. R. & Bank, R. A. Macrophage phenotypes in the collagen-induced foreign body reaction in rats. *Acta Biomater.* **9**, 6502–6510 (2013). 23376130 10.1016/j.actbio.2013.01.022

[35] Hishikawa, K., Oemar, B. S. & Nakaki, T. Static pressure regulates connective tissue growth factor expression in human mesangial cells. *J. Biol. Chem.* **276**, 16797–16803 (2001). 11278731 10.1074/jbc.M010722200

[36] Park, J. et al. Electromechanical cardioplasty using a wrapped elasto-conductive epicardial mesh. *Sci. Transl. Med.* **8**, 344ra86 (2016). 27334261 10.1126/scitranslmed.aad8568

[37] Choi, S. et al. Highly conductive, stretchable and biocompatible Ag-Au core-sheath nanowire composite for wearable and implantable bioelectronics. *Nat. Nanotechnol.* **13**, 1048–1056 (2018). 30104619 10.1038/s41565-018-0226-8

[38] Chavan, S. S., Pavlov, V. A. & Tracey, K. J. Mechanisms and therapeutic relevance of neuro-immune communication. *Immunity* **46**, 927–942 (2017). 28636960 10.1016/j.immuni.2017.06.008 PMC5578398

[39] Son, D. et al. An integrated self-healable electronic skin system fabricated via dynamic reconstruction of a nanostructured conducting network. *Nat. Nanotechnol.* **13**, 1057–1065 (2018). 30127474 10.1038/s41565-018-0244-6

[40] Veiseh, O. et al. Size- and shape-dependent foreign body immune response to materials implanted in rodents and non-human primates. *Nat. Mater.* **14**, 643–651 (2015). 25985456 10.1038/nmat4290 PMC4477281

[41] Song, K. I. et al. Compact optical nerve cuff electrode for simultaneous neural activity monitoring and optogenetic stimulation of peripheral nerves. *Sci. Rep.* **8**, 15630 (2018). 30353118 10.1038/s41598-018-33695-2 PMC6199280

[42] Jowett, N., Kearney, R. E., Know, C. J. & Hadlock, T. A. Toward the bionic face: a novel neuroprosthetic device paradigm for facial reanimation consisting of neural blockade and functional electrical stimulation. *Plast. Reconstr. Surg.* **143**, 62e–76e (2019). 30589784 10.1097/PRS.0000000000005164 PMC6311722

[43] Kato, K., Sawada, M. & Nishimura, Y. Bypassing stroke-damaged neural pathways via a neural interface induces targeted cortical adaptation. *Nat. Commun.* **10**, 4699 (2019). 31619680 10.1038/s41467-019-12647-y PMC6796004

[44] Eduok, U., Faye, O. & Szpunar, J. Recent developments and applications of protective silicone coatings: a review of PDMS functional materials. *Prog. Org. Caot.* **111**, 124–163 (2017).

[45] Gonzalez-Rivera, J., Iglio, R., Barillaro, G., Duce, C. & Tine, M. R. Structural and thermoanalytical characterization of 3D porous PDMS foam materials: the effect of impurities derived from a sugar templating process. *Polymers* **10**, 616 (2018). 30966650 10.3390/polym10060616 PMC6404115

[46] NATIONAL RESEARCH COUNCIL, Guide for the care and use of laboratory animals. (National Academies Press, 2010).

[47] Chu, J. U. et al. Improvement of signal-to-interference ratio and signal-to-noise ratio in nerve cuff electrode systems. *Physiol. Meas.* **33**, 943–967 (2012). 22551721 10.1088/0967-3334/33/6/943

[48] Percival D. B. et al. Wavelet methods for time series analysis. (UK: Cambridge University Press, 2000).

[49] Song, K. I., Chu, J. U., Park, S. E., Hwang, D. & Youn, I. Ankle-angle estimation from blind source separated afferent activity in the sciatic nerve for closed-loop functional neuromuscular stimulation system. *IEEE Trans. Biomed. Eng.* **64**, 834–843 (2017). 27323354 10.1109/TBME.2016.2580705

