# Supplementary material for: Adaptive self-healing electronic epineurium for chronic bidirectional neural interfaces
Source: Nat Commun. 2020 Aug 21;11:4195. doi: 10.1038/s41467-020-18025-3 (PMC7442836; doi:10.1038/s41467-020-18025-3)
Supplement: Supplementary file 1 — Supplementary Information [file 41467_2020_18025_MOESM1_ESM.pdf]

Supplementary Information

Adaptive self-healing electronic epineurium for chronic bidirectional neural interfaces

Kang-Il Song[1], Hyunseon Seo[1], Duhwan Seong, Seunghoe Kim, Ki Jun Yu, Yu-Chan Kim, Jinseok Kim, Seok Joon Kwon, Hyung-Seop Han, Inchan Youn*, Hyojin Lee*, Donghee Son*

*Correspondence to: *iyoun@kist.re.kr, hyojinlee@kist.re.kr, and daniel3600@g.skku.edu*

[1] These authors contributed equally to this work

This PDF file includes:

Supplementary Figures. 1-27

Supplementary Tables 1-3

Captions for Supplementary Video 1-5

Other Supplementary Materials for this manuscript include the following:

Supplementary Video 1-5

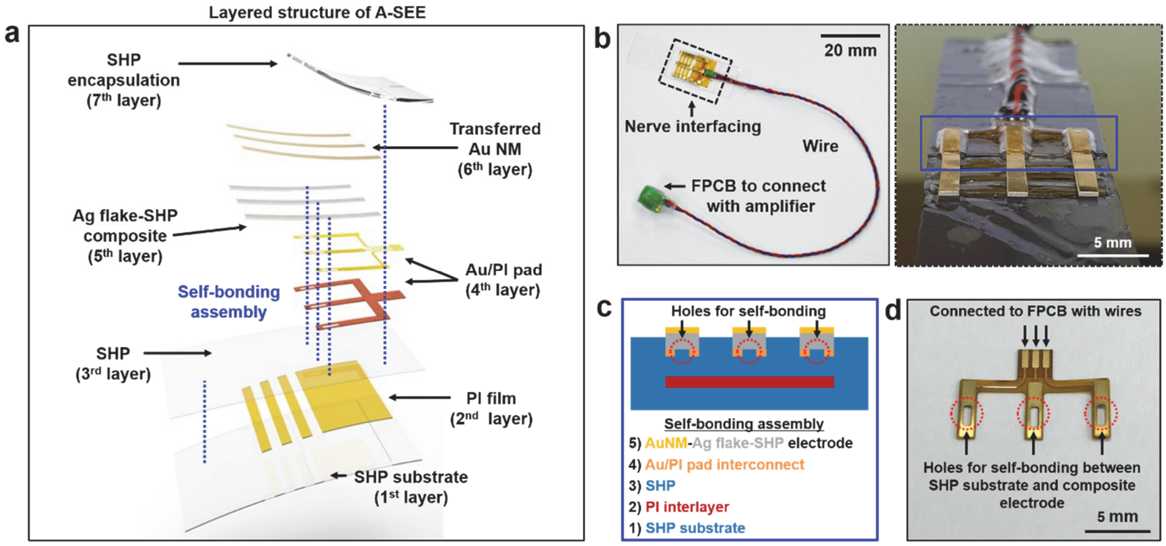

Supplementary Fig. 1. Structure of the A-SEE. a, Schematic showing the composition and layered structure of the A-SEE. The individual layers, including the substrate (SHP), electrode (AuNM-composite), interconnection for connecting with amplifier (Au/PI pad), interlayer for structural support (PI film), and encapsulation (SHP), could be easily integrated using the self-bonding assembly of self-healing materials (SHP and Ag flake-SHP composite) at room temperature. Specifically, the Au/PI pad (fourth layer) that interconnects between the AuNM-composite electrode and a flexible printed circuit board (FPCB) to connect with neural signal recording amplifier was placed on the SHP film (third layer: 35 mm long, 15 mm wide, and 0.25 mm thick). Next, three transferred AuNM-composite electrodes (fifth and sixth layer: 10 mm long, 1.5 mm wide, and 0.3 mm thick) were self-bonded to the SHP film (third layer) through the holes in the Au/PI pad (fourth layer). Then, the top of the electrode–pad integrated area was encapsulated by a SHP film (seventh layer: 9 mm long, 15 mm wide, and 0.1 mm thick). The length of the exposed electrode for the interfacing nerve was set to 6 mm. In addition, we introduced the PI film (second layer; Kapton tape, Dupont, 0.08 mm thick) as an interlayer between two SHP substrate films (first and third layers). This interlayer acted as a structural support for the SHP substrate and prevented wrinkling of the A-SEE that can be induced by substantial mechanical stress from surrounding muscles after implantation. Considering that the relatively stiff and rigid PI interlayer may increase the bending stiffness of the A-SEE, we designed three discrete PI films (11 mm long and 1 mm wide) that aligned with a 1 mm gap between them to facilitate the self-locking of the A-SEE. Furthermore, we confirmed that stress relaxation property of the A-SEE would not be reduced by the PI interlayer (Fig. 1c,d). **b**, Photographs showing the A-SEE connected to the FPCB with the interconnection wires. **c**, Schematic showing the cross-section of the A-SEE fabricated by the self-bonding assembly. **d**, Photograph of the Au/PI pad (fourth layer) and its holes for self-bonding.

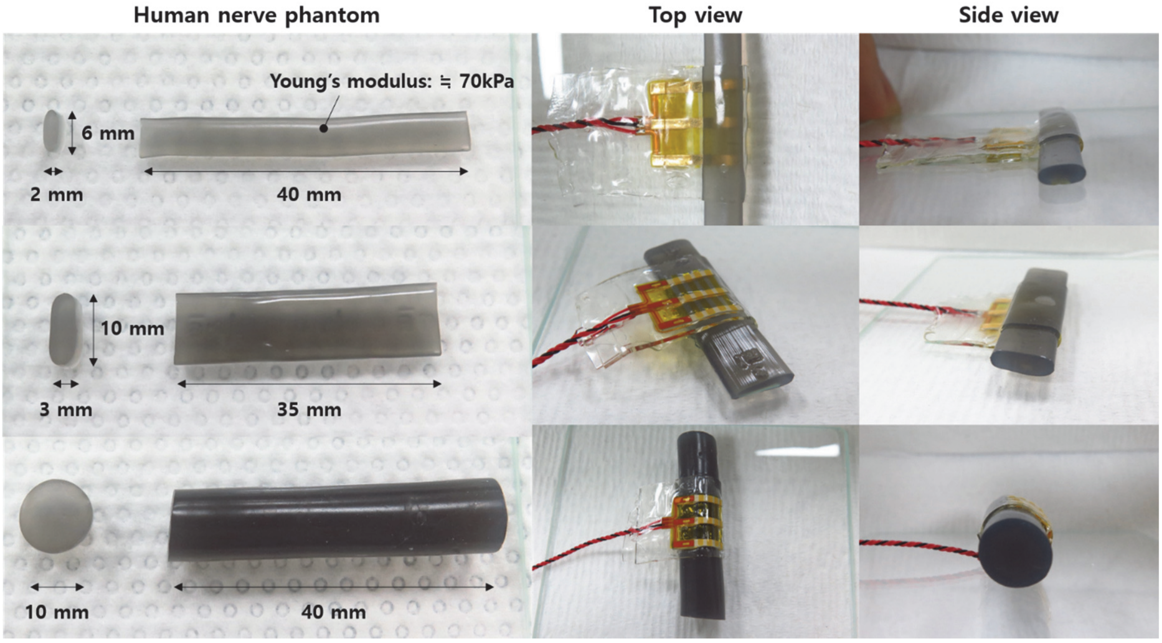

Supplementary Fig. 2. Mechanical and geometrical adaptability of the A-SEE to the human nerve phantom. The human nerve phantom was fabricated to mimic the various shapes of human nerves. The modulus of each plat shape nerve and round shape nerve is about 70 kPa. The mechanical and geometrical adaptability of the A-SEE enabled to form conformal interfaces without inducing any deformation to the human nerve phantoms.

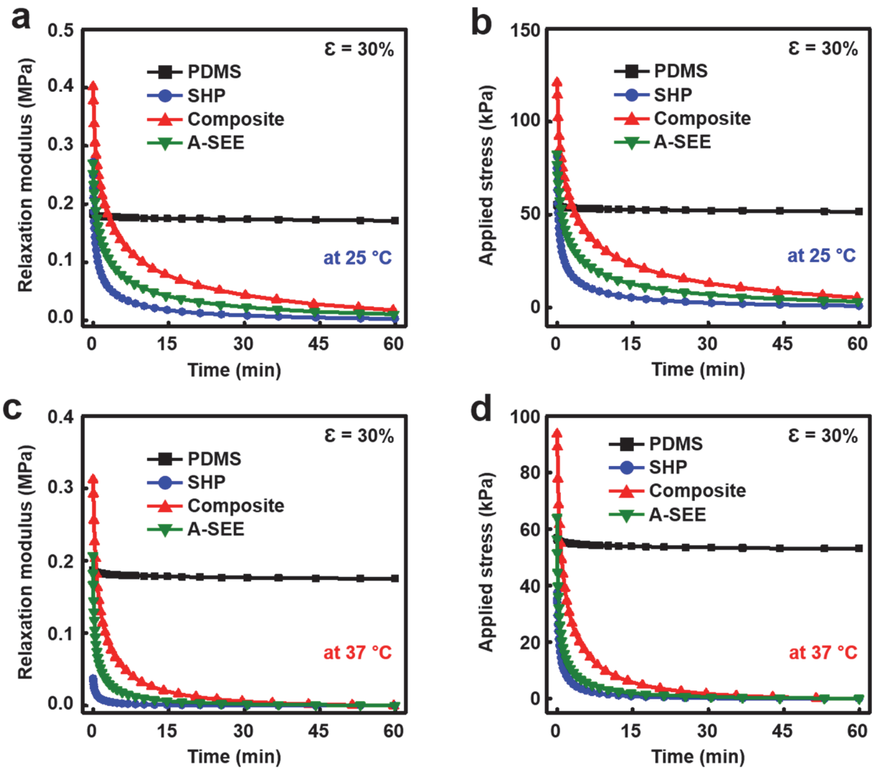

Supplementary Fig. 3. Time-dependent relaxation modulus and the applied tensile stress of the materials under 30% strain at 25 °C (a,b) and 37 °C (c,d). At 25 °C, the initial stress and the final stress after 1 h of relaxation under 30% tensile strain of the materials are 55 kPa and 52 kPa (PDMS), 81 kPa and 0.96 kPa (SHP), 121 kPa and 5.3 kPa (Composite), and 83 kPa and 3.3 kPa (A-SEE), respectively. Even though the initial applied stress of SHP and A-SEE were higher than that of PDMS, effective stress relaxation properties of SHP and A-SEE allowed to reach much lower stress than that of PDMS over time. At 37 °C, the initial stress and the final stress after 1 h of relaxation under 30% tensile strain of the materials are 57 kPa and 53 kPa (PDMS), 37 kPa and 0.003 kPa (SHP), 94 kPa and 3.6 kPa (Composite), and 64 kPa and 1.3 kPa (A-SEE), respectively. In addition to the stress relaxation performance, when the temperature increased, initial stress of SHP, composite, and A-SEE under 30 % strain significantly reduced owing to the thermoplasticity of SHP.

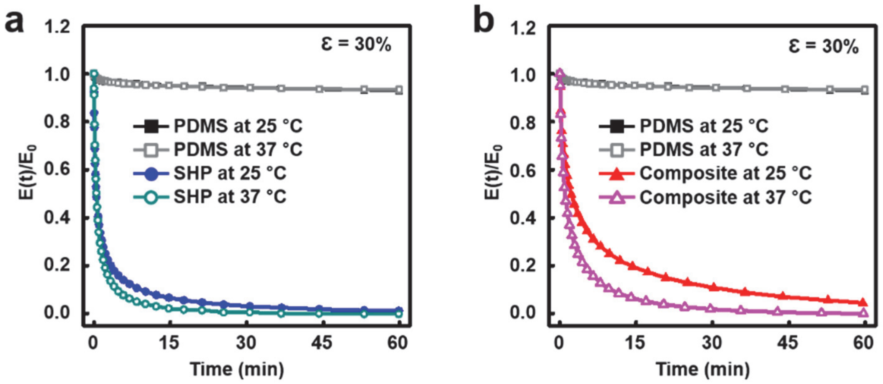

Supplementary Fig. 4. Temperature dependence of stress relaxation in SHP (a) and AuNM-Ag flake-SHP composite (b) compared to that in PDMS.

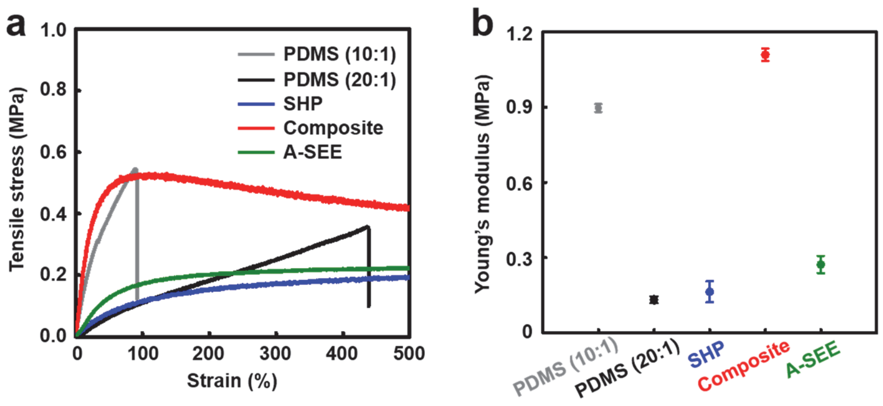

Supplementary Fig. 5. Stress-strain characteristics (a) and the calculated Young's modulus (b) of the materials. The 0.3 mm thick SHP film, the 0.3 mm thick AuNM-composite film, the 0.9 mm thick encapsulated AuNM-composite (A-SEE) film, and the 0.3 mm thick PDMS (Sylgard 184, PDMS:crosslinking agent weight ratio of 10:1 and 20:1) films were each cut to have a length of 10 mm and a width of 5 mm. Each sample was stretched at a strain rate of 50% per minute using Instron 5565 instrument. The calculated Young's modulus of the materials were 896.6 ± 16.0 kPa (PDMS with a PDMS:crosslinking agent weight ratio of 10:1), 131.8 ± 13.8 kPa (PDMS with a PDMS:crosslinking agent weight ratio of 20:1), 163.3 ± 42.1 kPa (SHP), 1109.0 ± 24.8 kPa (Composite), and 272.7 ± 34.5 kPa (A-SEE). We used PDMS with a PDMS:crosslinking agent weight ratio of 20:1 as the control material in all experiments due to its value of Young's modulus (131.8 ± 13.8 kPa) being comparable with that of SHP (163.3 ± 42.1 kPa).

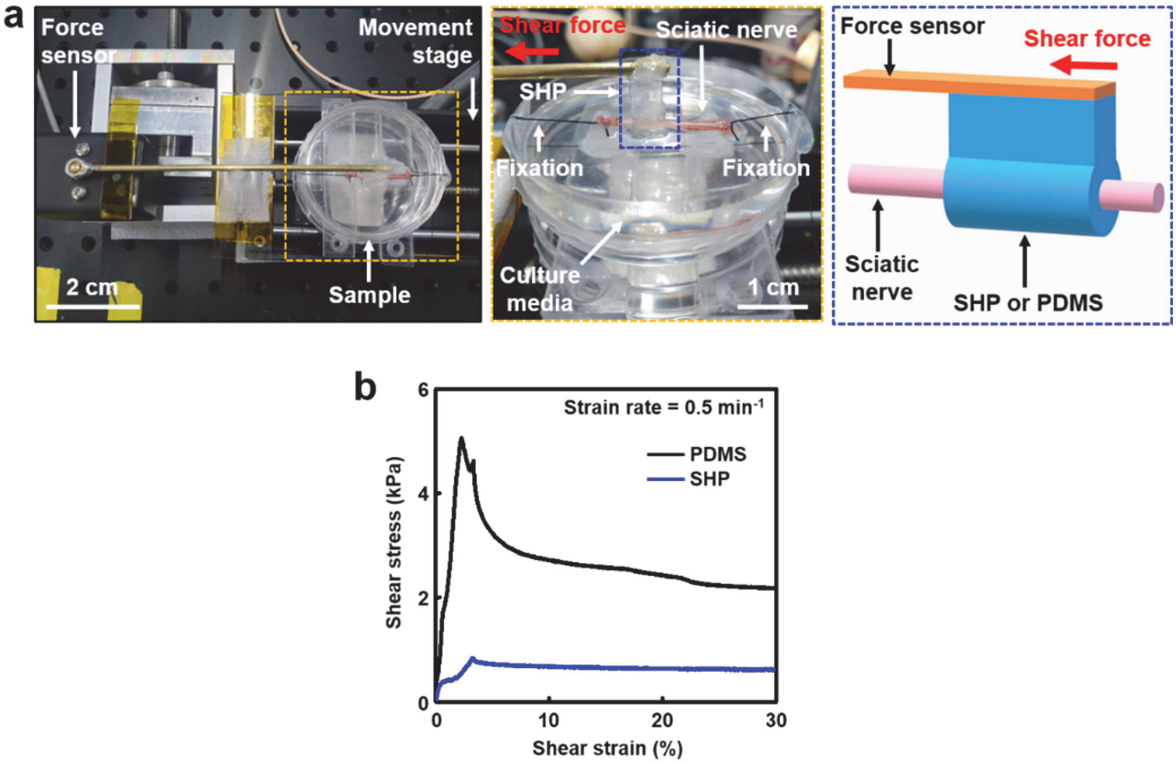

Supplementary Fig. 6. *Ex vivo* shear stress measurement set-up (a) and the measured shear stress-strain characteristics (b) of the materials. To examine the shear strength at the material-nerve interfaces induced by the movement of the nerve tissue, we designed an *ex vivo* shear force measurement set-up by using an isometric force transducer and an automatic unidirectional movement stage. A sciatic nerve tissue extracted from a rat was immersed in Krebs-Henseleit buffer solution (2000 mg/L Glucose, without calcium chloride and sodium bicarbonate) and fixed tightly by suturing. Then, the PDMS and SHP cuff structures which are connected to the force transducer were applied to the nerve tissue. The shear force was measured while the nerve tissue was moved by the automatic stage at a speed of 50% strain per minute. The measured maximum shear stress at PDMS-nerve interfaces and SHP-nerve interfaces was 5.06 kPa and 0.83 kPa, respectively, which are both much lower compared to the nerve tissue modulus (~100 kPa). After the maximum stress point, the stress values of ~2.2 kPa (PDMS) and ~0.6 kPa (SHP) were saturated, respectively. The remaining shear stress may indicate the kinetic friction between the materials and the nerve tissue in wet conditions. A much higher value of shear stress applied at PDMS-nerve interfaces (5.06 kPa) than that at SHP-nerve interfaces (0.83 kPa) may be due to the difference in the compressive stress induced by the fixed cuff structures. Effectively relaxed compressive stress at SHP-nerve interfaces facilitate the shear movement of the nerve tissue while maintaining conformal contact. Such a low level of the shear stress generated at SHP-nerve interfaces could be beneficial for safe and stable chronic implantation.

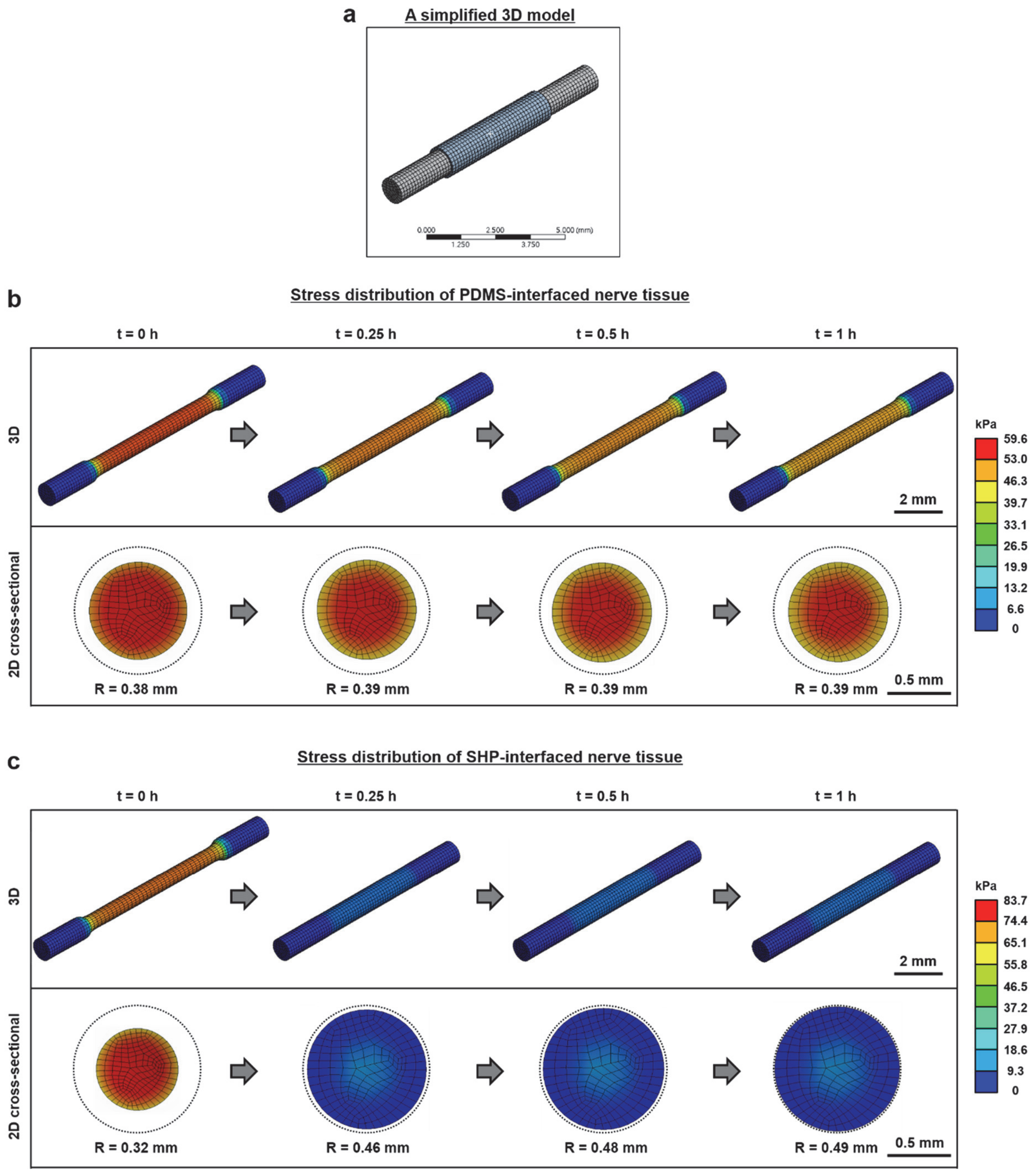

Supplementary Fig. 7. FEA 3D model (a) and the results showing the time-dependent distributions of compressive stress applied to the nerve interfaced with PDMS (b) and SHP (c).

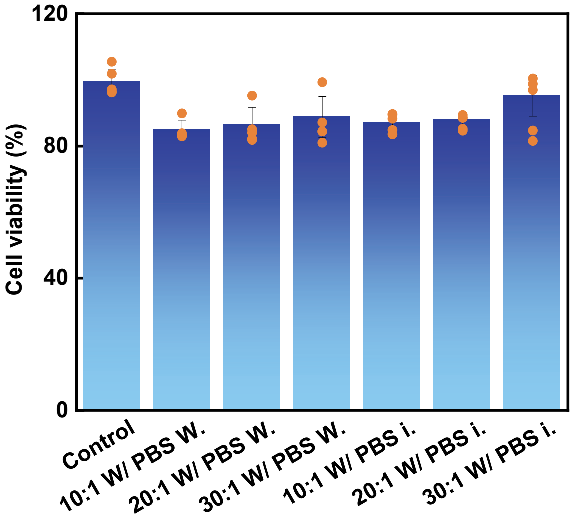

Supplementary Fig. 8. Cell viability test of PDMS samples sterilized by PBS washing (red) and PBS immersion (blue) methods. Owing to the potential toxicity of the remaining PDMS precursor, we compared two different PDMS sterilization methods. We first prepared the PDMS blocks with three different PDMS:crosslinking agent ratios (10:1, 20:1, and 30:1). Then, a group of PDMS was kept in PBS for 7 days with PBS replacement and the other group was immersed in 70% ethanol solution and washed twice with PBS right before the viability test[34,35]. We evaluated the cell viability 7 days after the incubation of cells with PDMS. We used a THP-1 cell that is a human monocyte cell grown in suspension, because the adherent cells are not able to attach to the surface of PDMS, which results in inaccurate viability. The result shows that the effect of the PDMS washing method on the cell viability is negligible. In all cases, the cell maintained a viability degree of over 85%, which means that all conditions are bio-compatible. Data are represented as mean ± S.D. (n=10^4 cells/mL examined over 5 independent experiments).

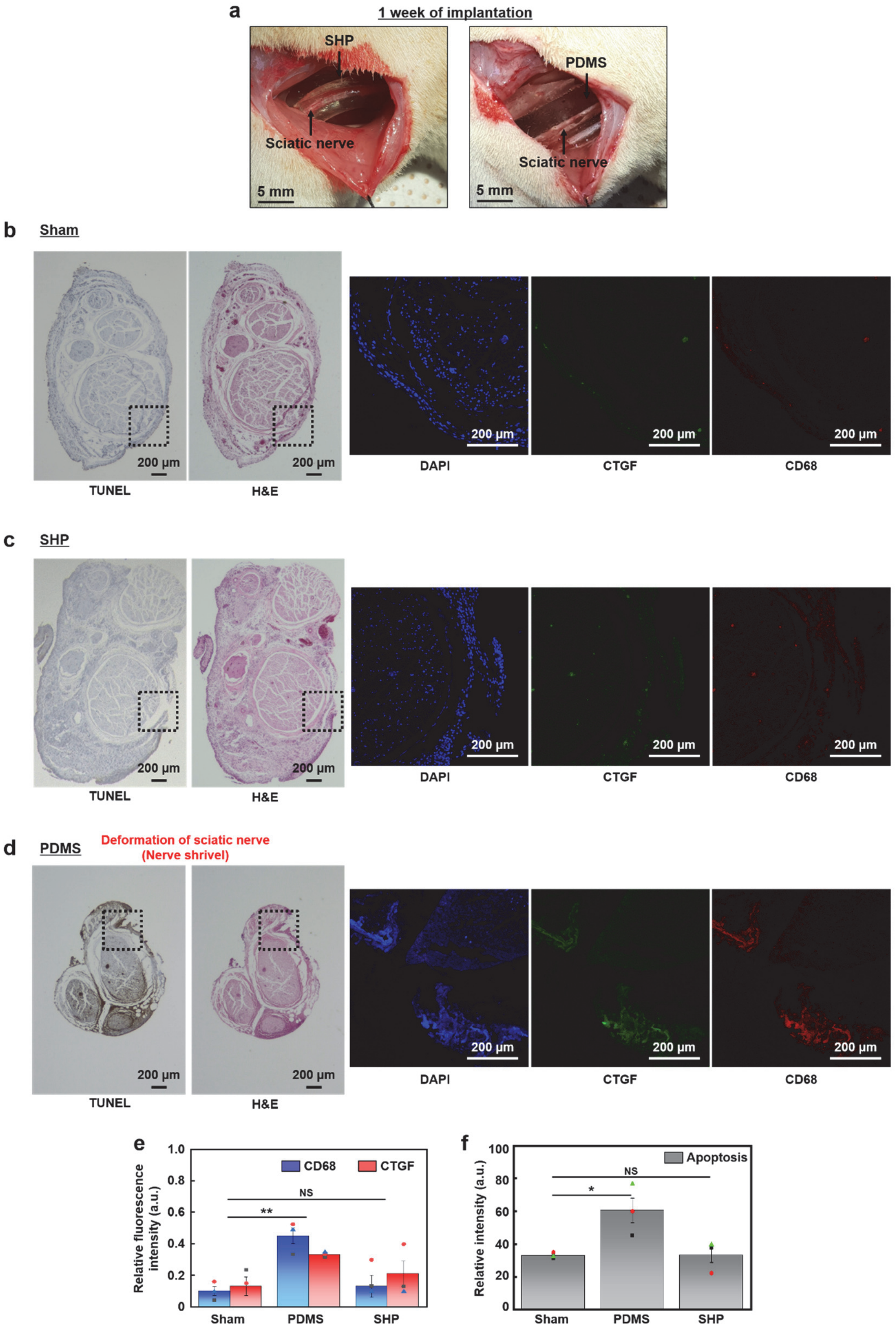

Supplementary Fig. 9. Histological and immunofluorescence analyses of rat sciatic nerve cross-sections after one week of SHP and PDMS implantation. a, Photographs of showing SHP (left) and PDMS (right) cuff structures implanted to rat sciatic nerves. **b-d**, TUNEL and H&E

staining images and immunofluorescence images of the rat sciatic nerves after one week of implantation (**b**, sham control; **c**, SHP; **d**, PDMS; Extended data for Fig. 1e-g). Representative images from n=3 biologically independent samples. H&E staining was used to confirm morphological changes and scar tissue formation. TUNEL assay was able to stain the apoptotic cells brown. **e**,**f**, Quantification of relative fluorescence intensity of CD68 and CTGF (**e**) and the relative intensity of TUNEL staining (**f**) from the immunostained nerve cross-section images shown in **b**-**d**. The results for H&E staining showed that the nerve tissue was deformed by PDMS while the nerve construct maintained in SHP. Furthermore, TUNEL staining results showed that PDMS cuff structure induced the apoptotic cell death due to the mechanical modulus mismatch at the biotic-abiotic interface. The immunofluorescence images for CTGF and CD68 represented that PDMS actively induced the CTGF over-expression and immune responses. Sham sample was used as a control (Dashed line means the areas for immunostaining images to magnify the tissue structures with cellular level scale.). In, **e**, **f**, all data are represented as mean ± S.D. Statistical analysis was performed using one-way analysis of variance (ANOVA) with Tukey's multiple comparison test (n=3 independent samples for each group, * P<0.05, ** P<0.01, and NS (not significant) P>0.05). For CD68 case in **e**, P(PDMS)=0.0005 and P(SHP)=0.5325. For CTGF case in **e**, P(PDMS)=0.0047 and P(SHP)=0.2381. For Apoptosis case in **f**, P(PDMS)=0.03 and P(SHP)=0.9149.

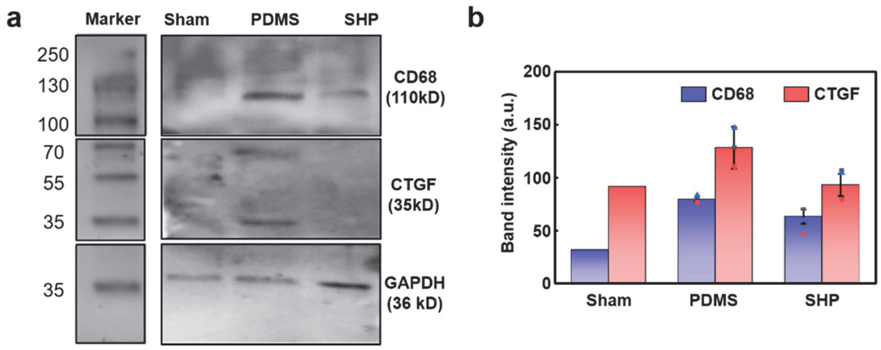

Supplementary Fig. 10. Immuno-blotting results for CD68 and CTGF after one week of PDMS and SHP implantation. a, CD68 and CTGF were detected by Western blotting to verify the effect of the PDMS and SHP stress relaxation properties on the changes in the protein expression levels of the interfaced nerve tissues. The GAPDH protein was used as a control protein. **b**, The average band intensity of CD68 and CTGF normalized by the GAPDH intensity value (n=3 independent samples). Both CD68 and CTGF expressions were highest in the PDMS-interfaced nerve tissue, which shows that continuous compressive stress induced by a mismatch in the mechanical modulus of PDMS and the nerve tissue resulted in overexpressed immune responses.

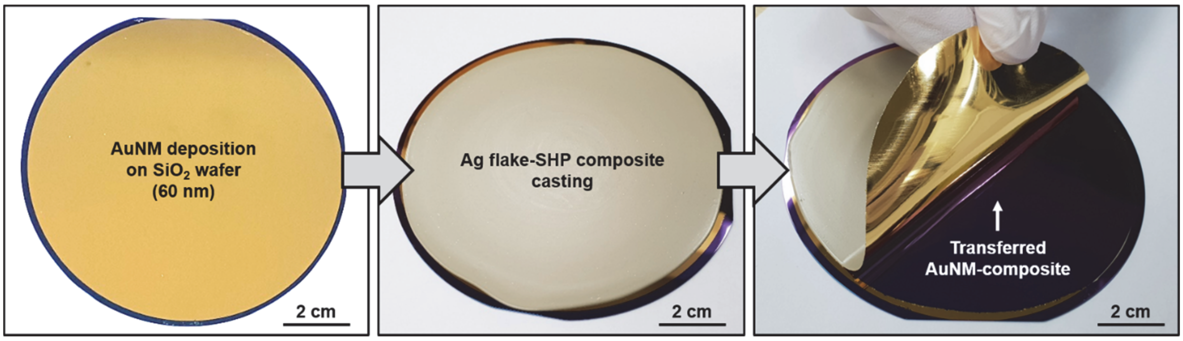

Supplementary Fig. 11. Fabrication of AuNM-Ag flake-SHP composite as an electrode of the A-SEE. Photographs showing the procedure of AuNM transfer onto the top surface of the Ag flake-SHP composite.

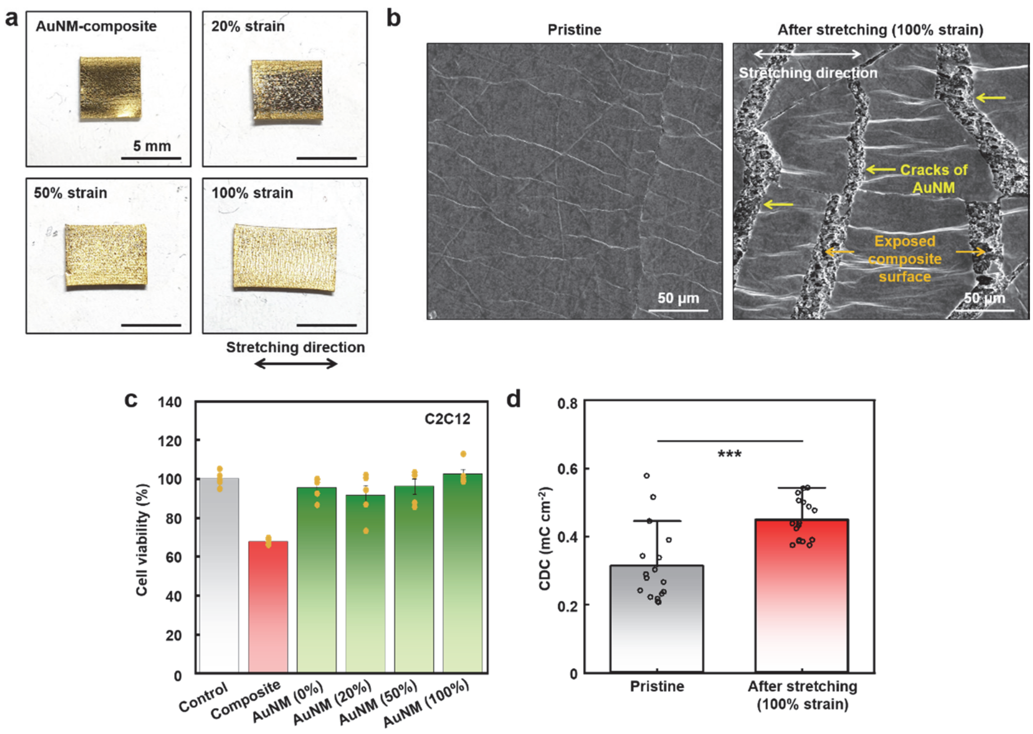

Supplementary Fig. 12. Cell viability and charge delivery capacity (CDC) of the stretched AuNM-composites. a, Photographs of AuNM-composite stretched with different tensile strains. **b**, SEM images of the AuNM-composite before and after stretching at 100% strain. Representative images from *n*=5 independent samples. **c**, Cell viability test after seven days culture. C2C12 cells were used for the test. The result clearly supports that cell death was not induced by leaked silver ions from the stretched AuNM-composite (**c**, green), compared to the composite sample with no AuNM (**c**, red). Data are represented as mean ± S.D. Statistical analysis was performed using one-way ANOVA with Tukey's multiple comparison test ($n=10^4$ cells/mL examined over 5 independent experiments. **d**, CDC values of the AuNM-composite before and after stretching at 100% strain. The CDC values were calculated from the obtained CV curves (20 cycles) shown in Fig. 2g (see Methods). Data are represented as mean ± S.D. Statistical analysis was performed using one-way ANOVA with Tukey's multiple comparison test (*n*=17 independent experiments, *P*=0.0001, *** P<0.001). When stretched to 100% strain, the CV curves shifted slightly (Fig. 2g) and the corresponding CDC values increased. These results may be correlated with cracks formed in the AuNM spreading to the surface of the composite. In the pristine state (before stretching), some AuNM wrinkles that may have been generated during the detachment from the oxide wafer were observed, but no cracks existed. However, at 100% strain state, cracks in the AuNM were generated because only the composite and SHP substrate were intrinsically stretchable and not the AuNM. Thus, the stretched electrode has a different surface nature compared with that of the pristine electrode due to the exposed Ag flake-SHP composite, which resulted in changes in the CV characteristics.

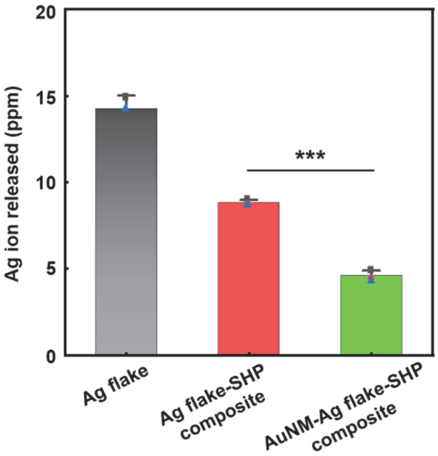

Supplementary Fig. 13. Stability test of the Ag flake, the Ag flake-SHP composite, and AuNM-Ag flake-SHP composite by quantification of Ag ions in cell culture medium. The amounts of Ag-ion leakage from the Ag flake, the Ag flake-SHP composite and the transferred AuNM-Ag flake-SHP composite immersed in DMEM for seven days were measured using ICP-MS. The significantly lower concentration of Ag ions released from the AuNM-Ag flake-SHP composite showed that AuNM can efficiently prevent Ag-ion leakage from the composite. These results confirm the role of the AuNM in enhancing the biocompatibility of the A-SEE by reducing the Ag-induced toxicity. Data are represented as mean ± S.D. Statistical analysis was performed using one-way ANOVA with Tukey's multiple comparison test (n=3 independent samples for composites and n=2 independent samples for Ag flake, P=0.0001, *** P<0.001).

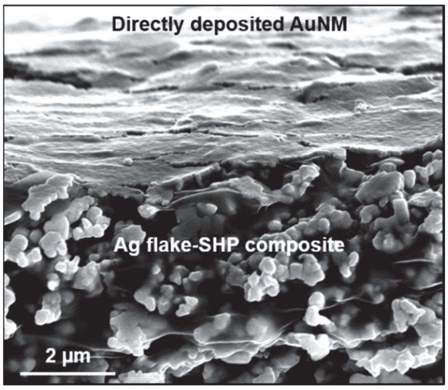

Supplementary Fig. 14. SEM image of the directly deposited AuNM-Ag flake-SHP composite. The AuNM could also be integrated with the Ag flake-SHP composite by direct e-beam deposition of the AuNM on the top surface of the Ag flake-SHP composite. However, compared to the transferred AuNM-composite (Fig. 2a), the surface of the deposited AuNM showed significant cracks. Representative image from *n*=5 independent samples.

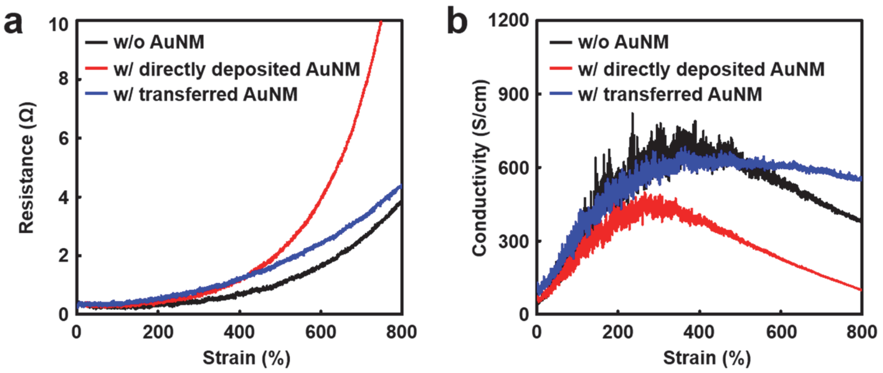

Supplementary Fig. 15. Comparison of electrical characterizations of Ag flake-SHP composites integrated with an AuNM using different processes. a,**b**, Resistance- (**a**) and conductivity- (**b**) strain curves of the Ag flake-SHP composites without AuNM (black), with directly deposited AuNM (red), and with transferred AuNM (blue) are shown. The electrical performance of the directly deposited AuNM-composite (357 S cm^{-1} at 200% strain and 99 S cm^{-1} at 800% strain) was much lower than that of the transferred AuNM-composite (502 S cm^{-1} at 200% strain and 558 S cm^{-1} at 800% strain). This difference in electrical performance may be due to 1) poor interfacial stability of the directly deposited AuNM with many cracks as shown in Supplementary Fig. 13, and 2) thermal degradation of the Ag flake-SHP composite during the deposition process[21].

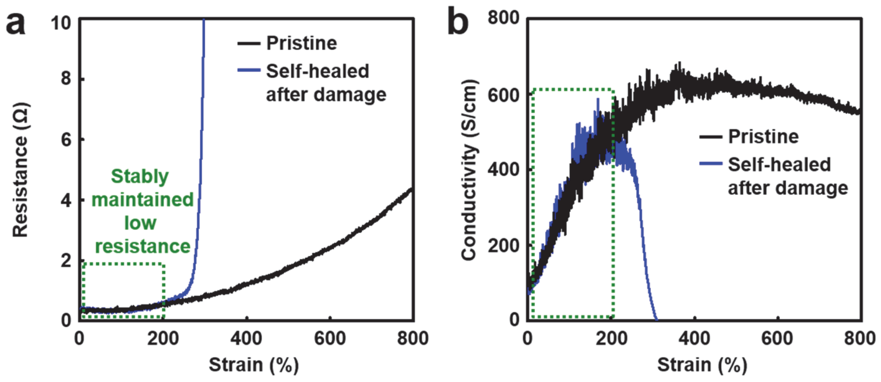

Supplementary Fig. 16. Self-healing of AuNM-Ag flake-SHP composite. a,**b**, Resistance- (**a**) and conductivity- (**b**) strain characteristics of the AuNM-Ag flake-SHP composite before damage (black) and after damage and self-healed (blue) are shown. The self-healed AuNM-composite after a complete cut showed comparable electrical performance up to approximately 200% strain. Such self-healing efficiency of the AuNM-Ag flake-SHP composite is similar to that of the composite without the AuNM shown in our previous report[21].

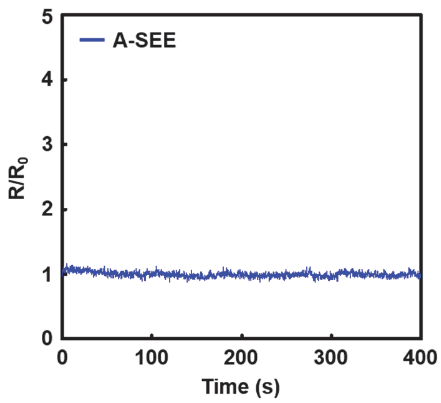

Supplementary Fig. 17. Electrical stability of the folded A-SEE in a nerve-electronics mimicking test. Normalized resistance of the A-SEE bent to the bending radius of a rat sciatic nerve (0.5 mm) are shown. The resistance of the A-SEE was constant while folded.

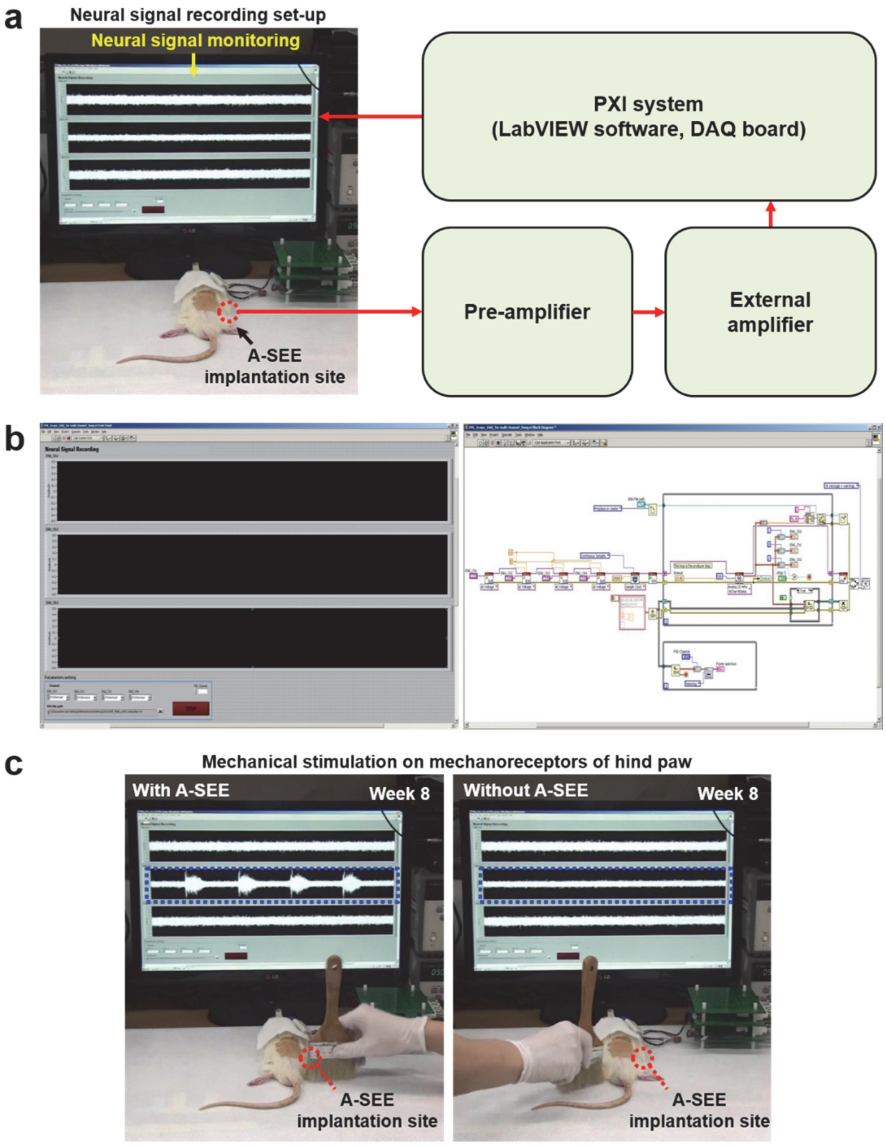

Supplementary Fig. 18. Experimental setup for sensory neural signal recording. a, Photograph and block diagram showing experimental setup for the sensory neural signal recording from a sciatic nerve of an anesthetized rat. **b**, Photographs showing graphic user interface (left) and LabVIEW program code (right) for neural signal monitoring. **c**, Photographs of the recorded sensory neural signals induced by mechanical stimulation. The recorded neural signals were displayed in the second row of the monitoring system (The first and the third row indicated the system noise of the analog-to-digital converter board). Since A-SEE was implanted to the right side sciatic nerve of the rat, the sensory neural signals could be recorded when the mechanical stimuli were applied on mechanoreceptors of the right side hind paw of the rat.

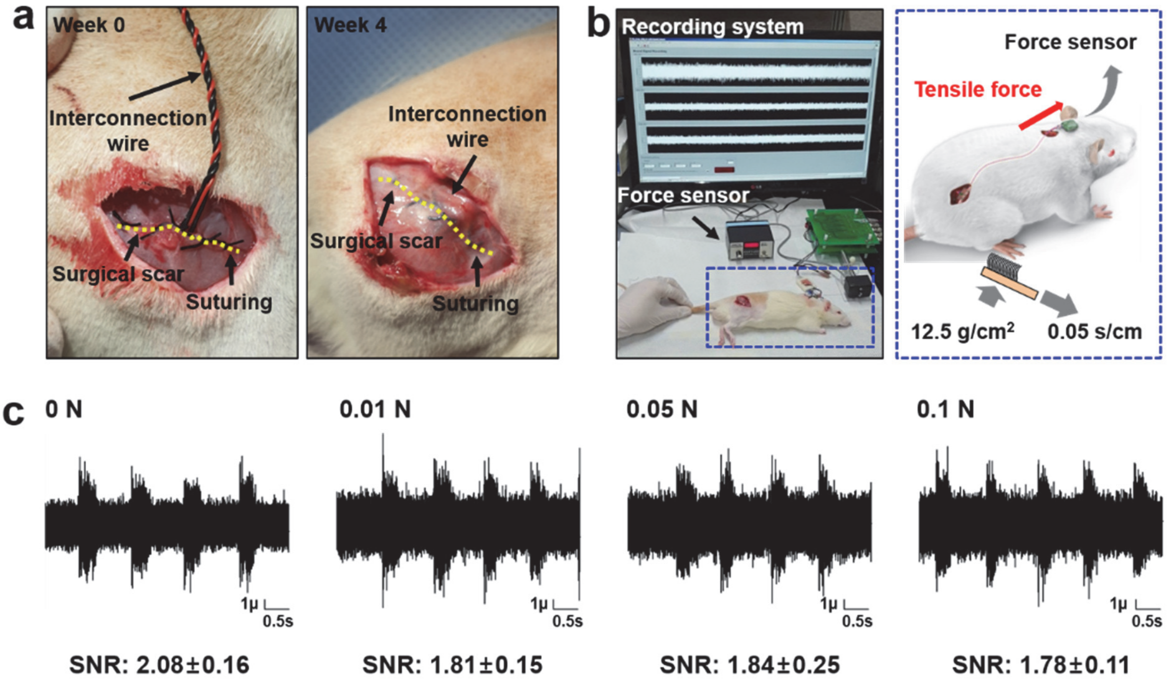

Supplementary Fig. 19. Mechanical stability of the A-SEE for neural signal recordings under the tensile force applied to the interconnection wire. a, Photographs showing surgical sites of A-SEE implantation after 0 and 4 weeks. **b**, Experimental setup for confirming the stability of neural signal recordings under tensile force applied to the interconnection wire. **c**, The recorded sensory neural signals from mechanical stimulation under different tensile forces (from 0.01 N to 0.1 N) applied to the interconnection wire after 4 weeks implantation of A-SEE. We could achieve stable neural signal measurement (SNR of 1.78 ~ 2.08) regardless of the applied force to the wire. The result shows that the self-bonding assembly of the A-SEE (see Supplementary Fig. 1) allows the contact interface to be electrically and mechanically passivated from external stimuli. In addition to our material uniqueness, the interconnection wire connected to our neural device was strongly fixed by wounded muscle.

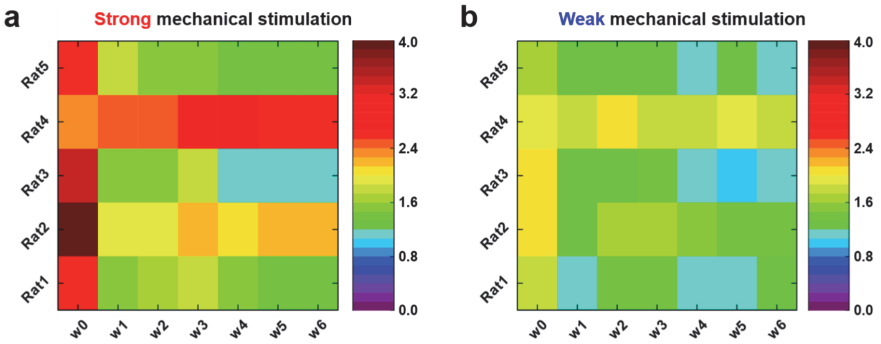

Supplementary Fig. 20. SNR color mapping of recorded neural signals for six weeks of implantation (n=5). a,**b**, The neural signals recorded from strong (**a**) and weak (**b**) mechanical stimulation during six weeks of the A-SEE implantation were shown as color mapping images. These color maps clearly show that the magnitudes of the recorded neural signals from the sciatic nerve become different depending on the mechanical stimulation induced on the hind paw.

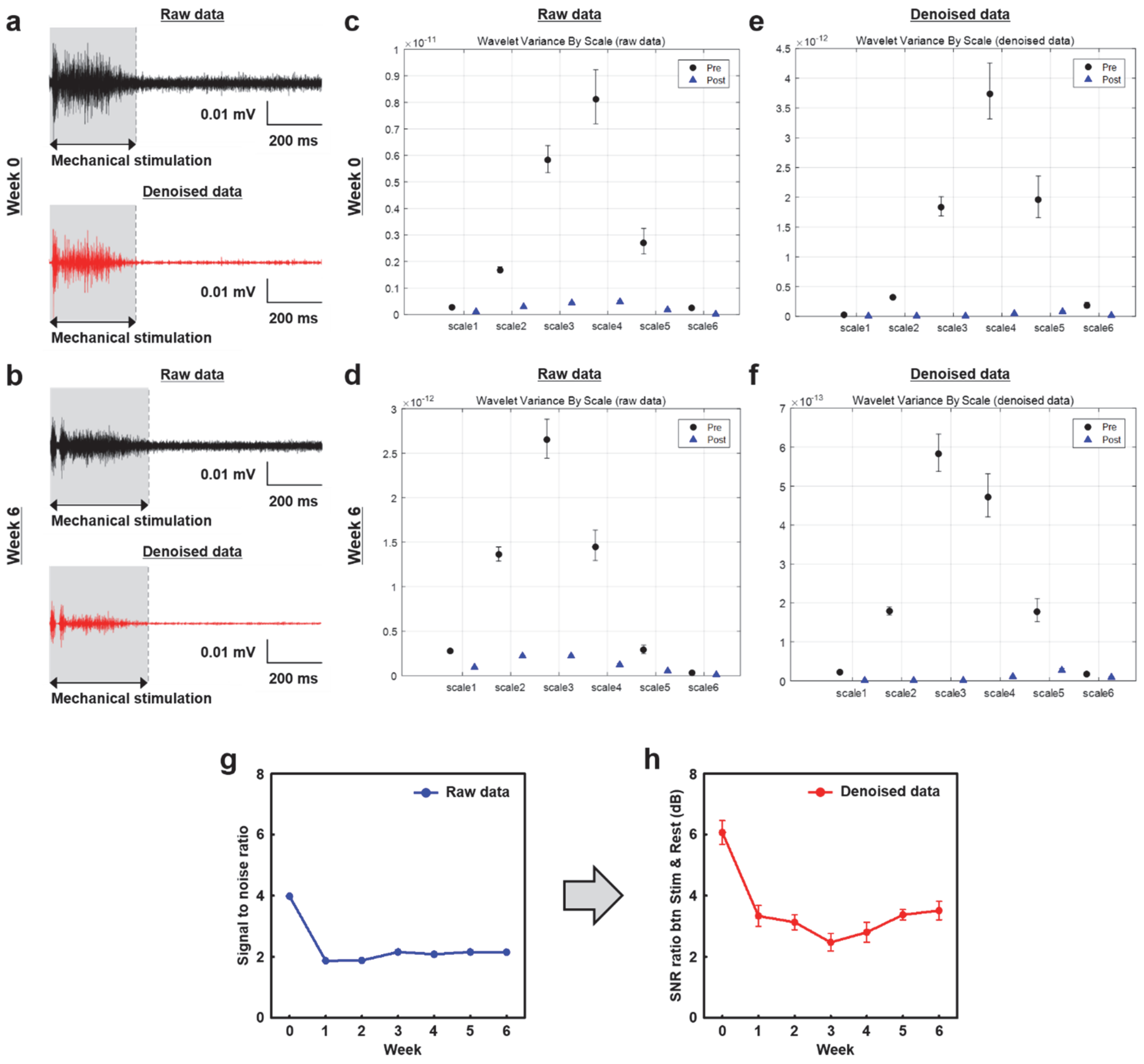

Supplementary Fig. 21. Denoising process of the recorded sensory neural signals. a,b, Raw signal data compared with denoised data for week 0 (**a**) and week 6 (**b**). **c-f**, Variance of the decomposed signals as a function of a time-window scale employed in the wavelet decomposition for the raw data (**c**,**d**) and the denoised data (**e**,**f**). **g**,**h**, The bottom two plots are the SNR of the raw data (**g**) and SNR ratio between the signals under stimulation and at the rest state (**h**), respectively. All data are represented as mean ± S.D. (*n*=5 data from independent animals).

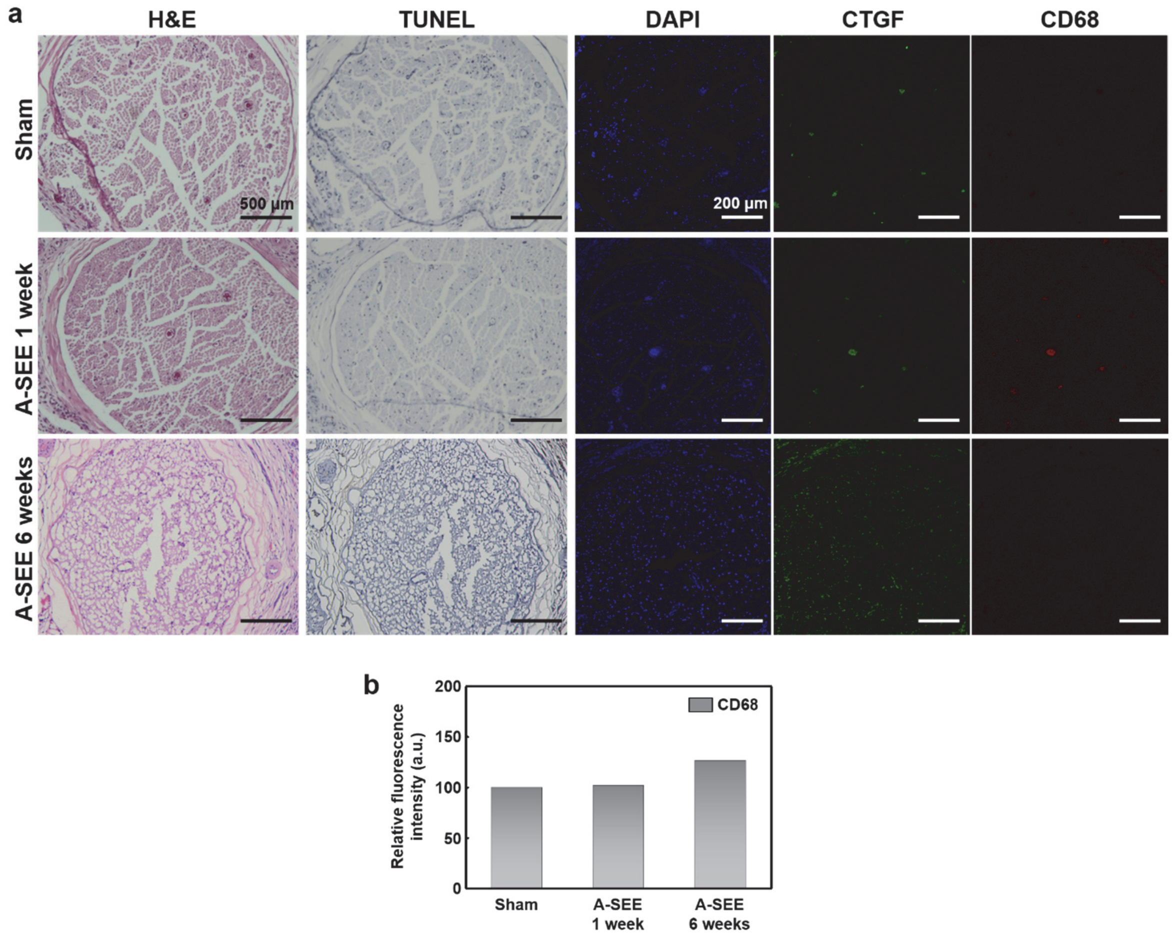

Supplementary Fig. 22. Histological and immunofluorescence analyses of rat sciatic nerve cross-sections after one week and six weeks of A-SEE implantation. a, H&E and TUNEL staining images and immunofluorescence images of the rat sciatic nerves after one week and six weeks of A-SEE implantation. Representative images from *n*=3 biologically independent samples. **b**, Quantification of relative fluorescence intensity of CD68 from the immunostained nerve cross-section images shown in (**a**). After one week of implantation, A-SEE-induced nerve tissue deformation and damages were negligible in H&E and TUNEL staining images. Additionally, CTGF (green) and CD68 (red) protein expression levels in the A-SEE implanted sample were similar to those observed in the sham control sample. After six weeks, the thickness of the fibrotic scar tissue around the nerve bundle slightly increased compared with the nerve tissue after one week. However, the cellular morphology and the nerve construct were well maintained, which indicates that the neurons functioned normally during long-term monitoring.

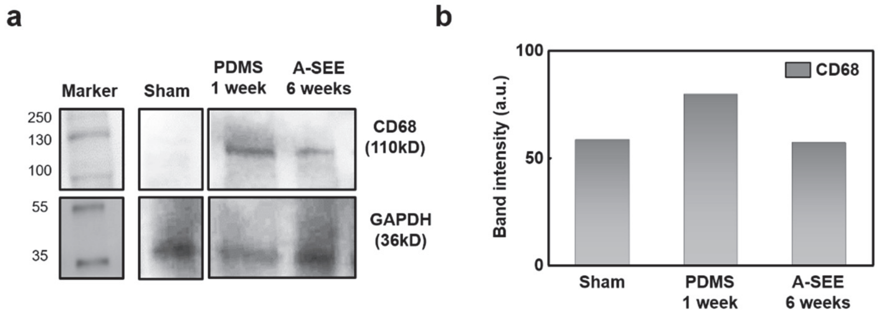

Supplementary Fig. 23. Immuno-blotting results for CD68 after one week of PDMS and six weeks of A-SEE implantation. a, CD68 was detected by Western blotting to verify the immune responses induced by 1 week implantation of PDMS and 6 weeks implantation of A-SEE. The GAPDH protein was used as a control protein. **b**, The average band intensity of CD68 normalized by GAPDH intensity value. The graph shows that normalized CD68 band intensity for 6 weeks A-SEE is almost the same as the CD68 level for sham sample. Importantly, the CD68 expression at 6 weeks implantation of A-SEE is relatively lower than the protein level for PDMS at only a week implantation. Based on the results, A-SEE could be the most promising one of the chronic peripheral neural interfaces.

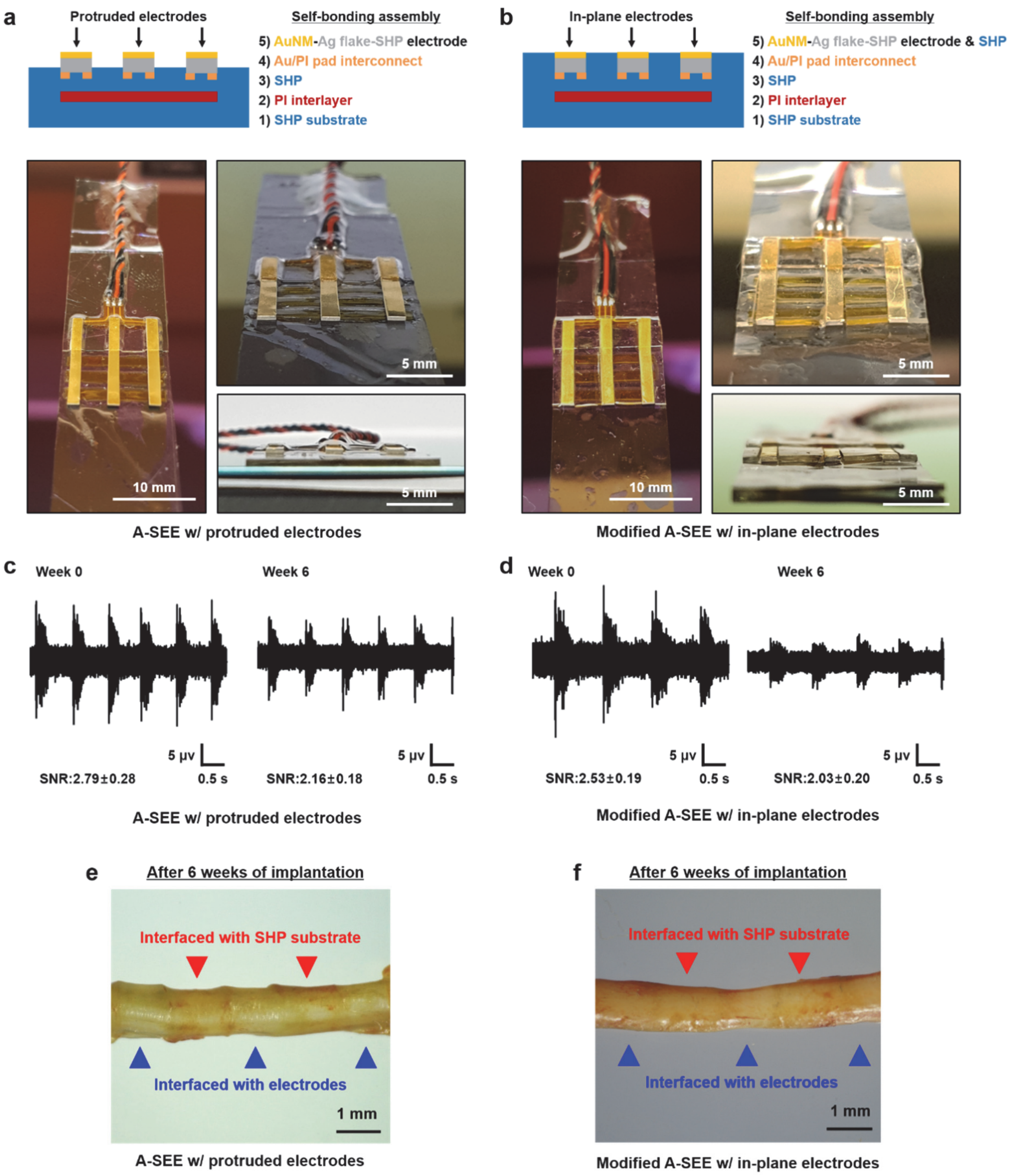

Supplementary Fig. 24. Comparison of the A-SEE with protruded electrodes (a, c, e) and the modified A-SEE with in-plane electrodes (b, d, f). The schematic and photographs showing the electrode structures (**a**, **b**), the recorded sensory neural signals (**c**, **d**), and the photographs of the explanted sciatic nerve interfaced with the A-SEEs for 6 weeks (**e**, **f**) are compared. We assumed that the small protruded fibrotic tissue formed at the SHP regions in the A-SEE with protruded electrodes (**e**) might originate from the physical height gap between the SHP regions and the nerve.

The composite electrodes of our neural interface were designed to be protruded on the SHP substrate (**a**), and thus SHP substrate regions between the electrodes might not be intimately contacted to the nerve tissue. Generally, any biological fluids can easily fill up the gap when implanted. The insufficiently contacted interfaces cause micro scar formation and the migration of immune-related cells, thereby resulting in the scar tissue formation (**e**). To better understand the complex interplay at the interface, we prepared a control experiment in which the A-SEE with a modified structure was fabricated to remove the protruding portion of the electrodes (**b**). We implanted the modified A-SEE to a rat sciatic nerve and recorded peripheral neural sensory signals for 6 weeks (**c**, **d**). As a result, we confirmed that the modified A-SEE could also achieve chronical stable neural signal recording. Furthermore, the explanted nerve tissue after 6 weeks of the modified A-SEE implantation showed no scar tissue formation at the whole interface areas (**f**), confirming our hypothesis.

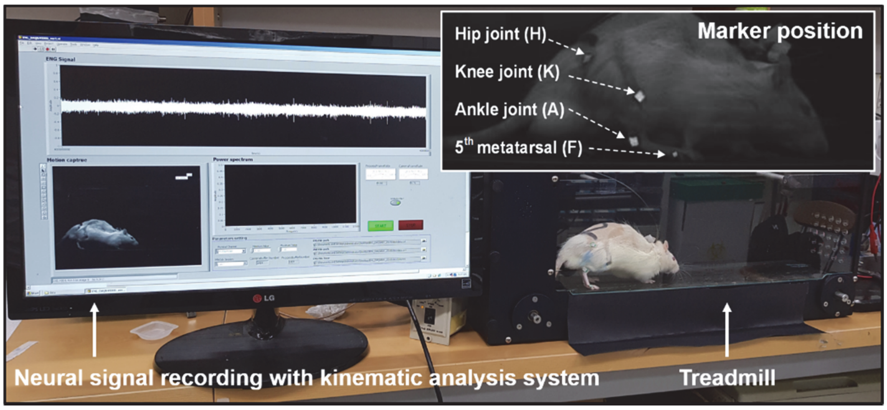

Supplementary Fig. 25. Simultaneous neural signal recording and joint kinematic analysis from an awake rat during treadmill walking. The joint kinematic analysis system consists of a neural signal recording amplifier, a high-speed digital camera, and a grabber board with control software. The digital camera was positioned perpendicular to the walking trace to record the images. The anatomical landmarks include the hip (H), knee (K), ankle (A), and fifth metatarsal (F) joints on the lateral side of the hind limb with the reflect marker positioned on the defined anatomical landmarks.

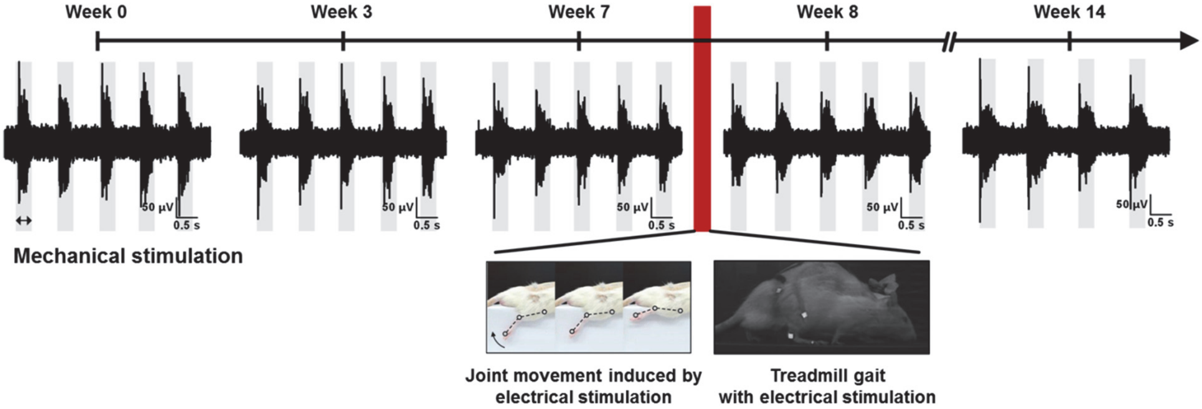

Supplementary Fig. 26. Chronic recordings of peripheral sensory neural signal. The sensory neural signals from sciatic nerve were successfully recorded after 14 weeks of A-SEE implantation (N=1, we did not sacrifice one of the implanted rats (N=5) up to now). Especially, the signals from a rat are recorded after repetitive electrical stimulation (over 200,000 pulses) for neuromodulation and treadmill gait experiments to confirm the stability for chronic bidirectional interfacing.

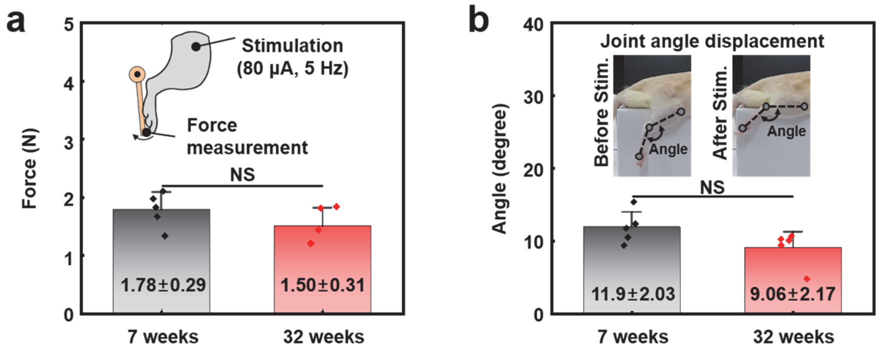

Supplementary Fig. 27. Electrical stimulation of a rat sciatic nerve interfaced with A-SEE for 32 weeks. The average force (**a**) and joint angle displacement (**b**) during electrical stimulation by A-SEE after 7 weeks and 32 weeks of implantation. Stimulation parameter was set to 80 μA with 5 Hz and 100 Hz for measuring force and joint angle displacement, respectively. The results showed the reliability of *in vivo* chronic neuromodulation performance of A-SEE. In **a**, **b**, data are represented as mean ± S.D. Statistical analysis was performed using one-way ANOVA with Tukey's multiple comparison test (n=5 independent experiments, P=0.18259 in **a**, P=0.0927 in **b**, NS (not significant) P>0.05).

Supplementary Table 1. Mechanical parameters of the materials and the nerve tissue used for FEA.

	Young's modulus (kPa)	Compressive stress at t = 0 h	Compressive stress at t = 0.25 h	Compressive stress at t = 0.5 h	Compressive stress at t = 1 h
PDMS	131.8	55.45	52.7	52.23	51.53
SHP	163.3	81.32	5.39	1.49	0.955
nerve[18]	100	.	.	.	.

Supplementary Table 2. Steps in the algorithm to calculate SNR of the recorded neural signal data.

Step	Description
1	Import raw data
2	Denoise the raw data using wavelet transform
3	Decompose the denoised signal using wavelet decomposition with various time scales
4	Calculate variance of the decomposed signal with various time scales
5	Find maximum variance change (gradient) point to define different types of the signal
6	Separate the two types of the signal based on the maximum variance change point
7	Calculate residual noise for each of the signal types (stim & rest)
8	Calculate signal-to-noise ratio (SNR) for each of the signal types
9	Calculate relative SNR ratio between the two signal types

Supplementary Table 3. The Au and Ag content in rat sciatic nerve tissues interfaced with the A-SEE for 6 weeks (Rat 1 & Rat 2) and 32 weeks (Rat 3), as measured by ICP-MS.

	Au	Ag
Rat 1 (6 weeks)	< 0.05	6.51
Rat 2 (6 weeks)	3.96	15.0
Rat 3 (32 weeks)	5.55	17.03

(ppm, mg/kg)

Supplementary Video 1. Self-locking process of A-SEE for interfacing the nerve.

Supplementary Video 2. Joint movements induced by electrical stimulation with different stimulations.

Supplementary Video 3. Treadmill gait in electrical stimulation.

Supplementary Video 4. Real-time neural signal recording at 0 week, 8 weeks, and 14 weeks implantation.

Supplementary Video 5. Demonstration of nerve-to-nerve interface.
