# Supplementary material for: Adaptive self-healing electronic epineurium for chronic bidirectional neural interfaces
Source: Nat Commun. 2020 Aug 21;11:4195. doi: 10.1038/s41467-020-18025-3 (PMC7442836; doi:10.1038/s41467-020-18025-3)
Supplement: Supplementary file 2 — Description of Additional Supplementary Files [file 41467_2020_18025_MOESM2_ESM.pdf]

Description of Additional Supplementary Files

File name: Supplementary Movie 1

Description: Self-locking process of A-SEE for interfacing the nerve.

File name: Supplementary Movie 2

Description: Joint movements induced by electrical stimulation with different stimulations.

File name: Supplementary Movie 3

Description: Treadmill gait in electrical stimulation.

File name: Supplementary Movie 4

Description: Real-time neural signal recording at 0 week, 8 weeks, and 14 weeks implantation.

File name: Supplementary Movie 5

Description: Demonstration of nerve-to-nerve interface.
