# Supplementary material for: Adaptive self-healing electronic epineurium for chronic bidirectional neural interfaces
Source: Nat Commun. 2020 Aug 21;11:4195. doi: 10.1038/s41467-020-18025-3 (PMC7442836; doi:10.1038/s41467-020-18025-3)
Supplement: Supplementary file 8 — Reporting Summary [file 41467_2020_18025_MOESM8_ESM.pdf]

nature research

Corresponding author(s): Inchan Youn, Hyojin Lee and Donghee Son

Last updated by author(s): Jul 29, 2020

Reporting Summary

Nature Research wishes to improve the reproducibility of the work that we publish. This form provides structure for consistency and transparency in reporting. For further information on Nature Research policies, see our Editorial Policies and the Editorial Policy Checklist.

Statistics

For all statistical analyses, confirm that the following items are present in the figure legend, table legend, main text, or Methods section.

n/a	Confirmed	
☐	☒	The exact sample size (n) for each experimental group/condition, given as a discrete number and unit of measurement
☐	☒	A statement on whether measurements were taken from distinct samples or whether the same sample was measured repeatedly
☐	☒	The statistical test(s) used AND whether they are one- or two-sided<br>*Only common tests should be described solely by name; describe more complex techniques in the Methods section.*
☐	☒	A description of all covariates tested
☐	☒	A description of any assumptions or corrections, such as tests of normality and adjustment for multiple comparisons
☐	☒	A full description of the statistical parameters including central tendency (e.g. means) or other basic estimates (e.g. regression coefficient) AND variation (e.g. standard deviation) or associated estimates of uncertainty (e.g. confidence intervals)
☐	☒	For null hypothesis testing, the test statistic (e.g. F, t, r) with confidence intervals, effect sizes, degrees of freedom and P value noted<br>*Give P values as exact values whenever suitable.*
☒	☐	For Bayesian analysis, information on the choice of priors and Markov chain Monte Carlo settings
☒	☐	For hierarchical and complex designs, identification of the appropriate level for tests and full reporting of outcomes
☒	☐	Estimates of effect sizes (e.g. Cohen's d, Pearson's r), indicating how they were calculated

Our web collection on statistics for biologists contains articles on many of the points above.

Software and code

Policy information about availability of computer code

Data collection	Labview was used for recording neural signals during mechanical stimulation, and treadmill movement. Origin was used for data plots.
Data analysis	To calculate the average signal of Western Blotting, each band intensity on gel was measured by Image J program (Rasband, W.S., S. National Institutes of Health, Bethesda, Maryland, USA). The P-value in all graphs were calculated using Origin 2020 softwear. The finite element analysis was performed using ANSYS Workbench (Release 16.1; ANSYS Inc. Canonsburg, PA, USA).

For manuscripts utilizing custom algorithms or software that are central to the research but not yet described in published literature, software must be made available to editors and reviewers. We strongly encourage code deposition in a community repository (e.g. GitHub). See the Nature Research guidelines for submitting code & software for further information.

Data

Policy information about availability of data

All manuscripts must include a data availability statement. This statement should provide the following information, where applicable:

- Accession codes, unique identifiers, or web links for publicly available datasets
- A list of figures that have associated raw data
- A description of any restrictions on data availability

All data used in this report are present in this report are present in the body of the report or in the online methods.

Field-specific reporting

Please select the one below that is the best fit for your research. If you are not sure, read the appropriate sections before making your selection.

☒ Life sciences ☐ Behavioural & social sciences ☐ Ecological, evolutionary & environmental sciences

For a reference copy of the document with all sections, see nature.com/documents/nr-reporting-summary-flat.pdf

Life sciences study design

All studies must disclose on these points even when the disclosure is negative.

Sample size	The neural signal was obtained from five Spargue Dawley rat's (300-350g, male). The number of animals was determined based on previous experiments. Ref: PubMed #30353118
Data exclusions	No data excluded from the viability test, neuromodulation, recording, and immunoblotting.
Replication	The neural signal recordings for mechanical stimulation were repeated more than ten times. The electrical stimulation were repeated more than ten times.
Randomization	All devices tested were selected randomly
Blinding	The investigators were blinded to to group allocation during data collection and analysis.

Reporting for specific materials, systems and methods

We require information from authors about some types of materials, experimental systems and methods used in many studies. Here, indicate whether each material, system or method listed is relevant to your study. If you are not sure if a list item applies to your research, read the appropriate section before selecting a response.

Materials & experimental systems

n/a	Involved in the study	
☐	☒	Antibodies
☐	☒	Eukaryotic cell lines
☒	☐	Palaeontology and archaeology
☐	☒	Animals and other organisms
☒	☐	Human research participants
☒	☐	Clinical data
☒	☐	Dual use research of concern

Methods

n/a	Involved in the study	
☒	☐	ChIP-seq
☒	☐	Flow cytometry
☒	☐	MRI-based neuroimaging

Antibodies

Antibodies used	Anti-GAPDH (ab9485, Abcam, Lot# GR3212164-2). anti-CD68 (ab53444, Abcam, Lot# GR3178467-2). anti-CTGF (ab6992, Abcam, Lot# GR2047-100), Goat Anti-Rabbit IgG (ab6721, Abcam, Lot# GR3196450-7), Goat Anti-Rat IgG (5C-2006, SantaCruz, Lot# G2514) Donkey Anti-Rat IgG H&L (Alexa Fluor® 647, Lot# GR-3188307-2) preadsorbed (ab150155), Goat Anti-Rabbit IgG H&L (Alexa Fluor® 488, Lot# GR-249483-2) (ab150077)
Validation	Anti-GAPDH: Host species (Rabbit), Tested applications Suitable for (IHC-P, IP, ELISA, WB, IHC-Fr, ICC/IF, Flow Cyt), Species reactivity Reacts with (Mouse, Rat, Chicken, Dog, Human, Saccharomyces cerevisiae, Xenopus laevis, Schizosaccharomyces pombe, African green monkey)<br>Immunogen (Full length native protein (purified) corresponding to Human GAPDH), Ref: PubMed #30341617<br>Anti-CD68: Host species (Rat), Specificity (ab53444 detects surface CD68 at low levels in resident mouse peritoneal macrophages which can be enhanced with thioglycollate stimulation), Tested applications Suitable for (ICC/IF, WB, IP, IHC-Fr, Flow Cyt), Species reactivity Reacts with (Mouse, Rat), Ref: PubMed #28553113<br>Anti-CTGF: Host species (Rabbit), Specificity (37-38 kDa, representing the CTGF protein, and at about 42-44 kDa likely representing a glycosilated or a phosphorylated form of CTGF), Tested applications Suitable for (IP, ICC/IF, WB, IHC-P, IHC-Fr, IHC-FoFr), Species reactivity Reacts with (Mouse, Rat, Sheep, Human), Ref: PubMed #30358017<br>Goat Anti-Rabbit IgG: Host species (Goat), Target species (Rabbit), Tested applications Suitable for: IHC-P, WB, ELISA, Immunomicroscopy, Dot blot, ICC, IHC-Fr), Immunogen (Rabbit IgG, whole molecule). Ref: PubMed # 30243997<br>Goat Anti-Rat IgG: goat anti-rat IgG-HRP is an affinity purified secondary antibody raised in goat against rat IgG and conjugated to HRP (horseradish peroxidase). Tested applications Suitable for (Western Blot, immunostaining, flow cytometry and ELISA), This antibody can be used to rat sample based on structure of epitope or expression level of targeted antibodies in rat tissue. Ref: PubMed # 18931023

Eukaryotic cell lines

Policy information about cell lines

Cell line source(s)	RAW 264.7 (ATCC® TIB-71™) and C2C12 (ATCC® CRL-1772™) cell lines were purchased form American Type Culture Collection (ATCC)
Authentication	Raw 264.7 and C2C12 were maintained in DMEM supplemented with 10% (v/v) fetal bovine serum, 100 U/ml penicillin/streptomycin (Thermo Fisher) at 37 °C with 5% CO2. Cells were purchased from ATCC and were authenticated and verified to be free of mycoplasma by ATCC.
Mycoplasma contamination	ATCC certified that the vials were not contaminated with mycoplasma. Additionally, we checked the cell morphology and culture dished using microscopy everyday during cell culturing.
Commonly misidentified lines (See ICLAC register)	We did not use any commonly misidentified cell lines in our experiments.

Animals and other organisms

Policy information about studies involving animals; ARRIVE guidelines recommended for reporting animal research

Laboratory animals	Sprague Dawley (SD) rat (300-350g, 10 week, male) were used in compliance with regulations of animal care and use committee of our institute. The experimental procedure was performed according to the Guide for the Care and Use of Laboratory Animals. The implantation surgery was performed under zoletil and xylize cocktail inhalation anesthesia.
Wild animals	The study did not involve wild animals.
Field-collected samples	The study did not involve samples collected form the field.
Ethics oversight	All animal experiments were performed and handled in accordance with the regulation of the Institutional Animal Care and Use Committee of the Korea Institute of Science and Technology (Approval No. 2018-067).

Note that full information on the approval of the study protocol must also be provided in the manuscript.
